# 3D Gelatin Microsphere Scaffolds Promote Functional Recovery after Spinal Cord Hemisection in Rats

**DOI:** 10.1002/advs.202204528

**Published:** 2022-12-01

**Authors:** Hongfei Ke, Hongru Yang, Yijing Zhao, Tingting Li, Danqing Xin, Chengcheng Gai, Zige Jiang, Zhen Wang

**Affiliations:** ^1^ Department of Physiology School of Basic Medical Sciences Cheeloo College of Medicine Shandong University 44 Wenhua Xi Road Jinan Shandong 250012 P. R. China; ^2^ State Key Laboratory of Crystal Materials Shandong University 27 Shanda Nanlu Jinan Shandong 250100 P. R. China

**Keywords:** axon regeneration, functional recovery, gelatin microspheres scaffolds, neuroinflammation, spinal cord injury

## Abstract

Spinal cord injury (SCI) damages signal connections and conductions, with the result that neuronal circuits are disrupted leading to neural dysfunctions. Such injuries represent a serious and relatively common central nervous system condition and current treatments have limited success in the reconstruction of nerve connections in injured areas, especially where sizeable gaps are present. Biomaterial scaffolds have become an effective alternative to nerve transplantation in filling these gaps and provide the foundation for simulating the 3D structure of solid organs. However, there remain some limitations with the application of 3D bioprinting for preparation of biomaterial scaffolds. Here, the approach in constructing and testing mini‐tissue building blocks and self‐assembly, solid 3D gelatin microsphere (GM) scaffolds with multiple voids as based on the convenient preparation of gelatin microspheres by microfluidic devices is described. These 3D GM scaffolds demonstrate suitable biocompatibility, biodegradation, porosity, low preparation costs, and relative ease of production. Moreover, 3D GM scaffolds can effectively bridge injury gaps, establish nerve connections and signal transductions, mitigate inflammatory microenvironments, and reduce glial scar formation. Accordingly, these 3D GM scaffolds can serve as a novel and effective bridging method to promote nerve regeneration and reconstruction and thus recovery of nerve function after SCI.

## Graphical abstract

3D gelatin microsphere scaffolds with suitable void size, biocompatibility, porosity, and degradability are fabricated by a microfluidic device with low cost and facile process. It is used to bridge injury gaps in rats. After spinal cord injury, its unique porous structure improves the microenvironment, promotes the survival and regeneration of neurons and axons, promoting functional recovery after spinal cord injury.

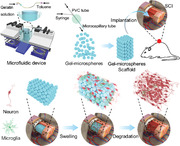

## 1 Introduction

Spinal cord injury (SCI) is a serious neurological condition which can readily lead to permanent disabilities or even death of patients along with exerting a heavy economic burden on both families and society.^[^ ^1^, ^2^ ^]^ Recovery of nerve function after SCI is realized through the regeneration and reconstruction of nerves and axons. However, in cases of severe tissue transections and/or serious damage, reconstruction of neuronal circuits requires a bridge to restore tissue continuity.^[^ ^3^ ^]^ In this way, restoration can be achieved, provided that the necessary physical support and guidance of the bridge enables regeneration and communication of the nerve stump to span these gaps.^[^ ^4^, ^5^ ^]^ Currently, repairs of injury gaps after SCI have involved autologous nerve transplantation. However, a number of limitations are associated with this frequently used clinical treatment, including donor site morbidity, mismatches in size and shape of the transplantation and limited tissue availability.^[^ ^6^ ^]^ In order to surmount these problems, widely sourced and strong, yet malleable, biomaterial scaffolds have gradually become an effective alternative to nerve transplantation, thus providing a new bridging strategy for the treatment of SCI.^[^ ^7^, ^8^, ^9^ ^]^

Effective spinal cord tissue function depends on a highly differentiated tissue architecture, with disruptions in this architecture producing severe deficits in neurological functions.^[^ ^10^ ^]^ Biomaterial scaffolds used to bridge and reconstruct damaged spinal cord tissue are often designed as nerve guided catheters which enable axonal extensions and directionally to guide the reinnervation.^[^ ^11^, ^12^ ^]^ However, a single tubular conduit structure is relatively ineffective for SCI repair as this structure may result in insufficient nutrient supply and oxygen/carbon dioxide exchange during nerve repair. These effects may then aggravate secondary injury processes associated with SCI,^[^ ^13^, ^14^ ^]^ such as inflammatory responses and scar formation.^[^ ^15^, ^16^ ^]^ Moreover, these single tubular conduit structures lack the ability to enable the complex 3D neural network of native nerves needed to support the multilevel crosstalk of neurons,^[^ ^16^, ^17^, ^18^ ^]^ thus resulting in limited nerve regeneration effectiveness. Such failings have prompted the development of new technologies directed toward designing biomaterial scaffolds with 3D porous structures for nerve regeneration,^[^ ^19^, ^20^, ^21^ ^]^ thereby facilitating the transition from single tubes to complex 3D solid organ construction.^[^ ^22^ ^]^ Currently, 3D bioprinting technology has involved simulating 3D structures of tissues and organs via a layered application of appropriate biomaterials while controlling the spatial layout.^[^ ^22^, ^23^, ^24^, ^25^, ^26^ ^]^ However, there remain some limitations with this application of 3D bioprinting for preparation of biomaterial scaffolds, including the high costs of equipment, limitations in printing precision, along with the time required for printing,^[^ ^27^, ^28^, ^29^, ^30^, ^31^, ^32^ ^]^ all of which hinder the application and promotion of clinical application. Accordingly, it is clear that an urgent need exists to produce biocompatible biomaterial scaffolds with low costs and ease of construction. Moreover, these scaffolds need to better reflect the anatomy and physiology of the spinal cord. In this way, they could create a suitable environment, in which the nerves receive sufficient nutrition and metabolic space and are effectively guided and protected to enable a successful regeneration and reconstruction process.

**Figure 1:**
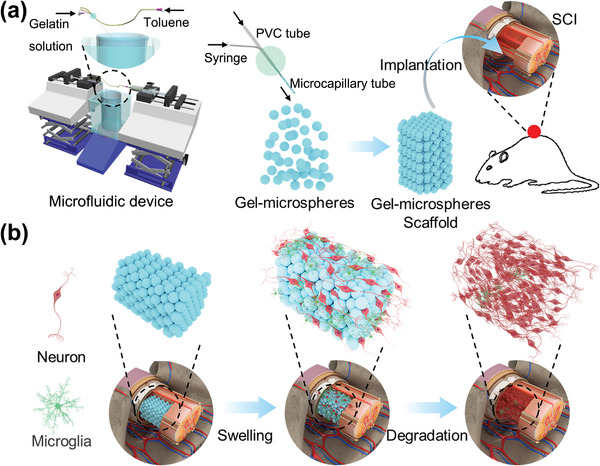
The 3D GM scaffolds for neuroregenerative medicine in SCI. a) Schematic illustration of the preparation of uniform gelatin microspheres and the assembly of 3D GM scaffolds. b) Schematic diagram of spinal cord repair and 3D GM scaffolds changes after implantation in the site of SCI.

In this report, we describe the production of 3D gelatin microsphere (3D GM) scaffolds with multiple voids as designed and fabricated on the basis of facile fabrication of gelatin microspheres via microfluidic devices. An effective production of such scaffolds can then be used to promote neurological recovery after SCI. The gelatin is obtained from collagen (a major component of the extracellular matrix) via partial hydrolysis.^[^ ^33^ ^]^ It is biocompatible, readily accessible, inexpensive and possesses a sufficient degree of plasticity.^[^ ^34^ ^]^ In our research, we applied the concepts of autonomous self‐assembly and mini‐tissue building blocks in 3D bioprinting,^[^ ^22^, ^35^ ^]^ to then prefabricate gelatin microspheres as mini‐tissue building blocks with relatively uniform diameters (215 ± 15 µm) that would be capable of promoting cell adhesion, growth and migration.^[^ ^36^, ^37^, ^38^ ^]^ Under the force of gravity, gelatin microspheres can be rapidly self‐assembled into 3D GM scaffolds (GMS) with large and uniform voids, to provide a suitable amount of mechanical strength (460 kPa).^[^ ^39^, ^40^ ^]^ This type of 3D structure also permits a balance between the biodegradability and the support required during SCI treatment to enable a match between 3D GM scaffold degradation rates and degree of nerve regeneration.^[^ ^41^ ^]^ The unique porous structure of these 3D GM scaffolds provides the structural basis for nerve regeneration, thus simulating the anatomical and physiological structural reconstruction processes that most closely mimic that of spinal cord tissue.^[^ ^39^, ^41^, ^42^, ^43^ ^]^ Our results demonstrate that these 3D GM scaffolds bridged the lesion gap, alleviated ulcerative edema and cystic vacuoles, and promoted the regeneration of spinal cord tissue. Simultaneously, 3D GM scaffolds promoted the survival and regeneration of nerves and axons after SCI by inhibiting microglial activation and astrocyte proliferation as well as by reducing the expression of proinflammatory factors and glial scar formation. These findings establish 3D GM scaffold implantations as a method which can promote the recovery of neurological function after SCI and provide a new and effective strategy for the treatment of SCI (**Figure** **1**).

## 2 Results and Discussion

### 2.1 Characterization of 3D GM Scaffolds

**Figure 2:**
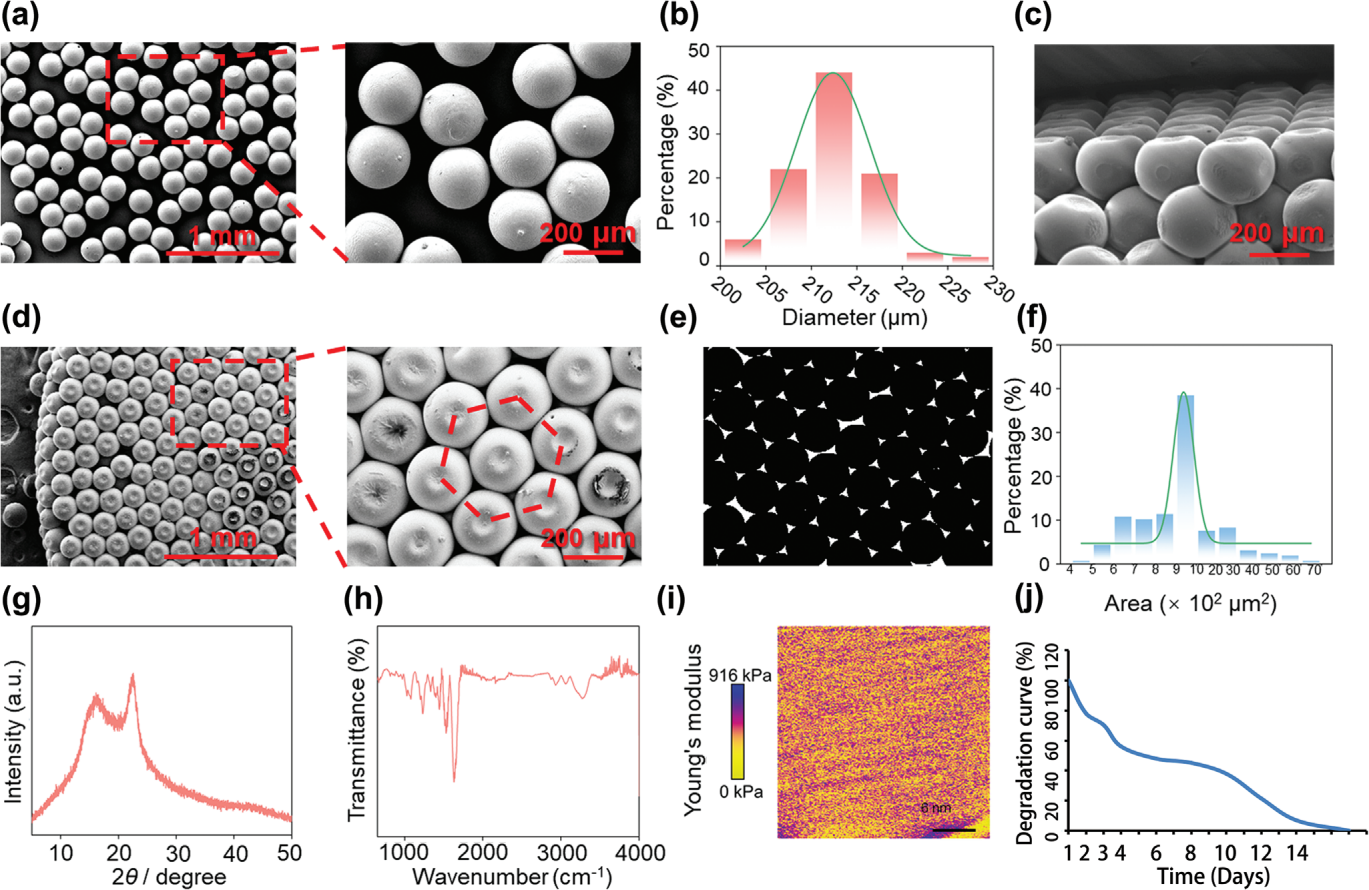
The characterization of morphologies, compositions, and mechanical properties of 3D GM scaffolds. a) SEM images of the obtained gelatin microspheres. b) The particle size distribution of the obtained gelatin microspheres. c) The side‐view SEM image of 3D GM scaffolds. d) The top‐view SEM image of 3D GM scaffolds. e) Contrast diagram of void and microsphere area obtained through Image J transformation. f) The void area distribution of the 3D GM scaffolds. g) XRD patterns of the 3D GM scaffolds. h) FTIR spectra of the 3D GM scaffolds. i) Young's modulus analysis for 3D GM scaffolds. j) Degradation curve of 3D GM scaffolds.

Based on concepts derived from autonomous self‐assembly and mini‐tissue building blocks in 3D bioprinting, gelatin microspheres were prefabricated as mini‐tissue building blocks and self‐assembled into cubic close packed (ccp) gelatin microsphere scaffolds by gravity. The gelatin microspheres were produced from gelatin, as a type of hydrophilic and natural polymer, using a simple fluidic device fabricated with a poly vinyl chloride (PVC) tube, a syringe needle and a glass capillary tube as described previously. By adjusting flow rates, viscosity, density, surface tension and channel geometry, the discontinuous phase was completely surrounded by the continuous phase enabling the final construction of gelatin microspheres. These microspheres were thoroughly dispersed, remarkably uniform in size, maintained their shapes as demonstrated with scanning electron microscope (SEM) (**Figure** **2**a), and measured 215 ± 15 µm in diameter as determined using Image J software (Figure 2b). This size has been demonstrated to be optimal for cell adhesion, growth, and migration.^[^ ^38^, ^43^ ^]^

When driven by gravity, gelatin microspheres rapidly self‐assembled into ccp solid 3D GM scaffolds in a centrifuge tube. A side‐view SEM image of the final results, as obtained following further heat curing and freeze‐dried molding, is shown in Figure 2c. Due to solvent evaporation, gelatin microspheres may show a certain degree of shrinkage during scaffold assembly. A top‐view SEM image of ccp solid 3D GM scaffolds is shown in Figure 2d. The 3D GM scaffolds consisted of a regular arrangement of gelatin microspheres. There was an abundance of voids in these 3D GM scaffolds, which are necessary for SCI repair as they serve as pathways for cell migration and nutrient/waste transport. In contrast to this complex 3D structure simulating that of the native spinal cord, nerve guidance conduits with single tubular structures are relatively ineffective in peripheral nerve bridging for nerve connection and motor function recovery in SCI repair.^[^ ^44^, ^45^ ^]^ In order to measure the area of void in each layer of the 3D GM scaffolds, Image J software was used to transform the top‐view of the SEM image, with the contrast diagram of the void and microsphere area shown in Figure 2e and the void area distribution of the 3D GM scaffolds in Figure 2f. It was found that nearly 90% of the void areas were between 6 and 30 × 10^2^ µm^2^ in these 3D GM scaffolds (Figure 2f). Ideal scaffolds should allow for a high degree of interconnectivity and a suitable void size to enhance the transport of nutrients/metabolic wastes and prevent void occlusion during tissue formation.^[^ ^14^, ^38^ ^]^ Most previous attempts on scaffold construction have been less effective in achieving survival and regeneration of cells as they failed to possess the specific condition as described above.^[^ ^16^, ^46^ ^]^ X‐ray diffraction (XRD) patterns of these 3D GM scaffolds are shown in Figure 2g, with the result that only two peaks at 2*θ* ≈ 15° and ≈23° were obtained, which was consistent with the *α*‐helices and *β*‐sheets of gelatin, respectively. Fourier transform infrared spectroscopy (FTIR) spectra of these 3D GM scaffolds are presented in Figure 2h. The spectra of samples revealed characteristic peaks at 1645 cm^−1^ as corresponding to amide I (CO stretching), 1535 cm^−1^ for the amide II band (CN stretching and NH bending) and 1242 cm^−1^ for amid III (CH stretching and NH bending). These results all verified that the 3D GM scaffolds were completely composed of gelatin (Figure 2h). Mechanical property analysis of 3D GM scaffolds indicated that the scaffolds exhibited an average Young's modulus of around 460 kPa (Figure 2i), which was similar to that as observed in normal spinal cords (200–600 kPa).^[^ ^39^, ^40^ ^]^ Such a resemblance effectively prevents a mechanical mismatch between scaffolds and the spinal cord, thus decreasing the potential for additional physical injury. The degradation characteristics results showed that the 3D GM scaffolds degraded gradually with time after reaching swelling equilibrium (4 h), and completely degraded over time. The equilibrium volume was about five times larger than the initial volume (Figure S2 and Table S2, Supporting Information). It degraded to about 80% of the equilibrium volume on days 2 and about 50% on the days 7. It will degrade to 20% of the equilibrium volume until the days 14 and completely degraded after days 17 (Figure 2j and Figure S3 and Table S3, Supporting Information). To prove the biocompatibility of 3D GM scaffolds, the exogenous mesenchymal stem cells were cultured on 3D GM scaffolds in vitro, the results showed that mesenchymal stem cells can well adhere and migrate on the 3D GM scaffolds (Figure S4, Supporting Information).

As shown in Figure S5a in the Supporting Information, control gelatin scaffolds (GS) were cured as a solid gelatin block without a 3D structure. In these control gelatin scaffolds no voids were found as determined by transforming the SEM image as obtained with use of Image J software (Figure S5b, Supporting Information).

### 2.2 3D GM Scaffolds Promote Behavioral Recovery Following Spinal Cord Hemisection

**Figure 3:**
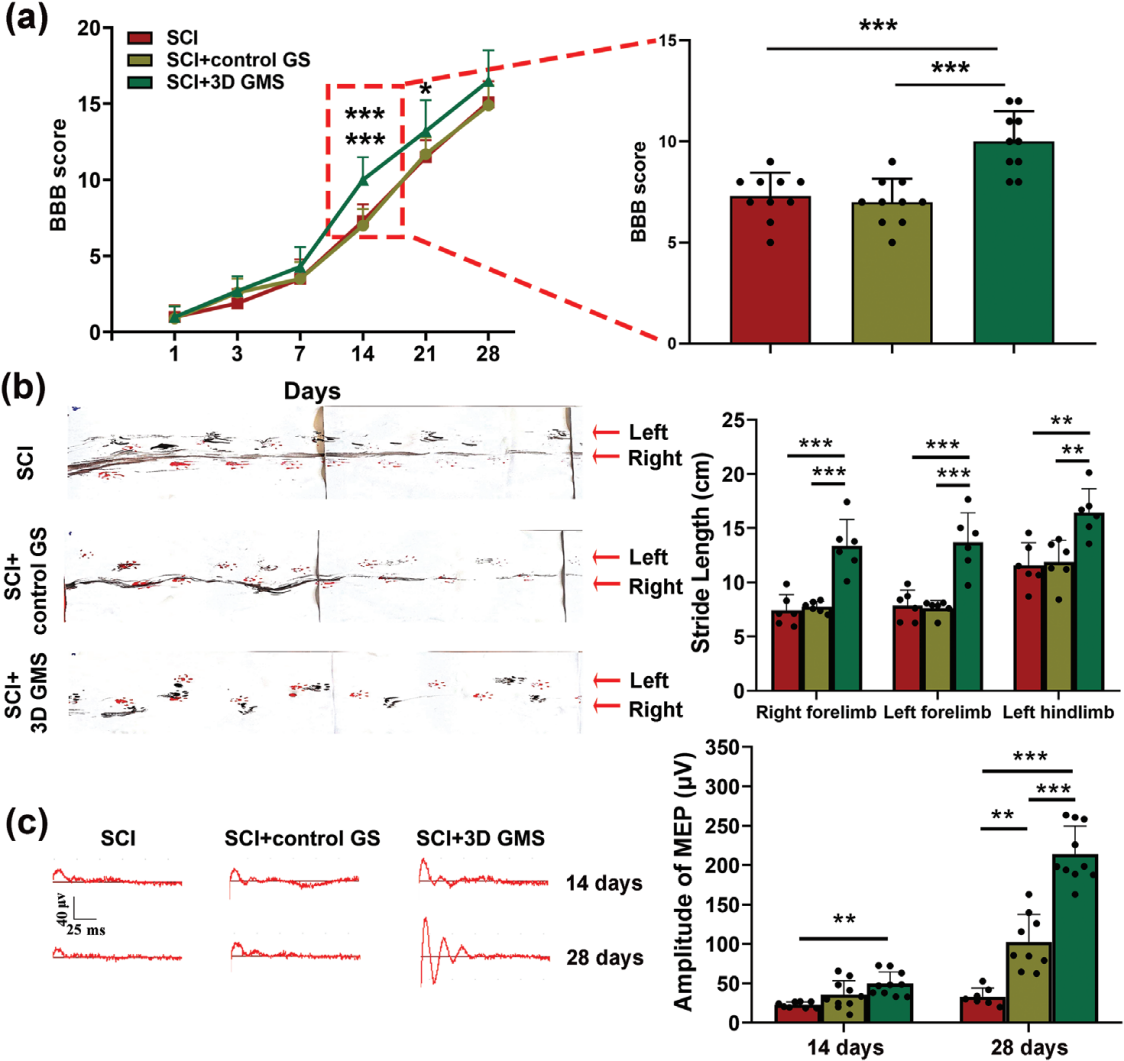
3D GM scaffolds promoted neurological function recovery at 28 d after surgery. a) BBB limb function scores at different times at 1, 3, 7, 14, 21, and 28 d after surgery. *N*  =  10 rats per group. b) Typical footprints of animal walking at 28 d after SCI. The forelimb footprints were shown in blue and the hindlimb footprints were shown in red. Statistical analysis of the stride of right forepaw, left forepaw, and left hindpaw. *N*  =  6 rats per group. c) Typical images of right hindlimb motor evoked potentials (MEP) in the three groups at 14 and 28 d after surgery. Statistical analysis of the amplitude of right hindlimb MEP. *N*  =  8 rats per group. Values represent the mean ± standard deviations (SD), *p < 0.05, **p < 0.01, ***p < 0.001 according to ANOVA.

Behavioral recovery was first evaluated using the Basso, Beattie, and Bresnahan (BBB) rating scale (Figure S6, Supporting Information). A two‐way analysis of variance with one repeated measure was conducted to assess the effect of treatment (no construct, control GS, and 3D GMS) and time (days post‐SCI) on BBB scores. Significant main effects were obtained for time, with increases in motor function recovery observed over the period from days 1 to 28 [*F*(6108) = 2450.407, *p* < 0.001] as well as for treatment [*F*(2108) = 4.359, *p* < 0.05]. Specifically, at 1, 3, and 7 d postinjury, there were no significant differences in BBB scores among the three groups, but significant functional recoveries were observed in the 3D GM scaffolds versus SCI group on days 14 [*F*(2,27) = 16.714, *p* < 0.001; post hoc *p* < 0.001] and 21 [*F*(2,27) = 4.005, *p* < 0.05; post hoc *p* < 0.05] (**Figure** **3**a). Moreover, statistically significant differences were obtained between the control GS versus 3D GMS group on days 14 (post hoc *p* < 0.001; Figure 3a and Videos S1–S3, Supporting Information), while no significant differences were present between the control GS and SCI group (Figure 3a). As shown in Figure S7b in the Supporting Information, extensive movement of all three hind limb joints was observed in SCI + 3D GMS rats, whereas those in the SCI and SCI + control GS groups continued to drag their hind legs with little improvement in hip joint movement at 14 d after injury. Footprint analyses at 28 d post‐SCI demonstrated that the SCI group showed a significant reduction in the coordination of their right limb movements. Rats in the SCI + control GS groups showed a slight gait recovery but rats treated with 3D GMS showed a significantly more rapid gait recovery and improved motor coordination versus the SCI and SCI + control GS groups [left forelimb: 13.70 ± 2.72 vs 7.86 ± 1.42 and 7.62 ± 0.70 cm, *F*(2,15) = 24.278, *p* < 0.001; right forelimb: 13.36 ± 2.44 vs 7.42 ± 1.45 and 7.73 ± 0.50 cm, *F*(2,15) = 21.497, *p* < 0.001; left hindlimb: 16.45 ± 2.20 vs 11.58 ± 2.06 and 11.87 ± 2.03 cm, *F*(2,15) = 10.176, *p* < 0.01] (Figure 3b).

Before surgery, stable waveforms of motor evoked potentials (MEP) were recorded in the left/right hindlimb of the three groups (Figure S8, Supporting Information, and Figure 3c). At 14 d postinjury, statistically significant increases in MEP amplitudes were obtained in the SCI + 3D GMS (49.41 ± 15.05 µV) as compared with that of the SCI group (22.64 ± 3.71 µV) [*F*(2,25) = 8.045, *p* < 0.01; post hoc *p* < 0.01]. However, MEP amplitudes of the SCI + control GS group (35.74 ± 17.78 µV) were not significantly different (post hoc *p* > 0.05) from the SCI group (Figure 3c).

Further increases in MEP amplitudes were observed at 28 d postinjury with these amplitudes in the SCI + 3D GMS group (213.88 ± 35.88 µV) now being significantly greater than those in both the SCI (33.13 ± 11.05 µV) and SCI + control GS (102.27 ± 35.27 µV) groups [*F*(2,23) = 73.669, *p* < 0.001; post hoc *p* < 0.001] (Figure 3c). These results indicated that the 3D GM scaffolds exerted a beneficial therapeutic effect on functional motor responses in rats following SCI.

### 2.3 3D GM Scaffolds Improve Lesion Repair Following Spinal Cord Hemisection

**Figure 4:**
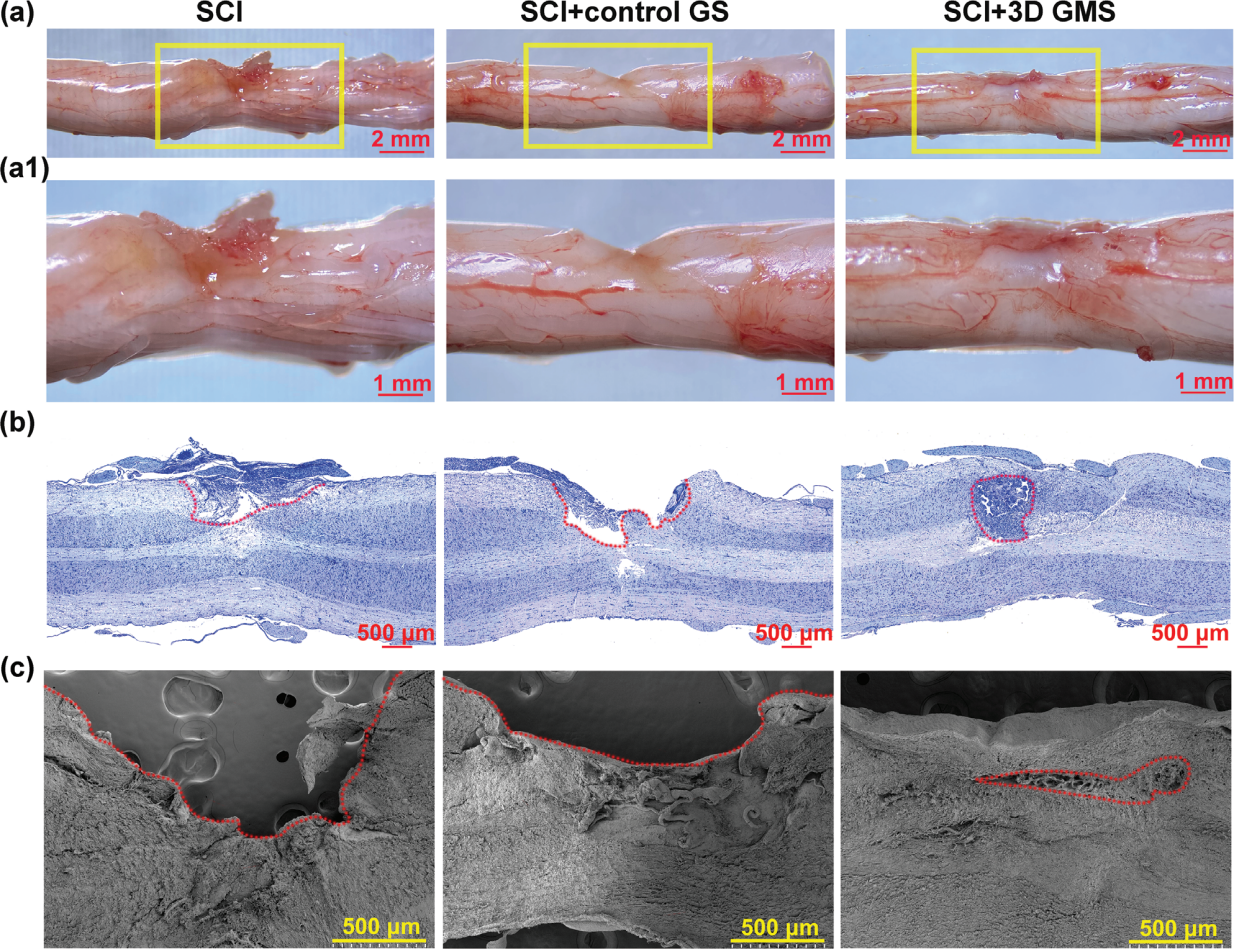
3D GM scaffolds attenuated tissue loss at 28 d after surgery. a) General views of spinal cord tissue at the injury site in three groups. Scale bar = 2 mm. a1) Magnification of yellow frames in (a). Scale bar = 1 mm. b) Nissl staining of spinal cord tissue. The cavity boundary was indicated by the dashed lines. Scale bar = 500 µm. c) SEM of spinal cord tissue. The cavity boundary was indicated by the dashed lines. Scale bar = 500 µm.

Spinal cord tissue function depends on a highly differentiated tissue architecture, such that disruptions in this architecture result in severe deficits in neurological function.^[^ ^10^ ^]^ Nerve disruptions in SCI cannot be directly sutured,^[^ ^47^, ^48^ ^]^ therefore scaffolds are required to bridge the injury gap and provide a suitable microenvironment for tissue regeneration. At 28 d postinjury, macroscopic observations indicated that edema, ulceration, tissue defects, and cystic cavities were present in damaged tissue within the SCI and SCI + control GS groups, changes which were clearly different from that observed in the periphery of normal tissues (**Figure** **4**a,a1). In contrast, the SCI + 3D GMS group showed a good degree of connectivity and integration between regions of the injured spinal cord and surrounding normal tissues (Figure 4a,a1). It seems that the abundance of voids present within the 3D structure of the GM scaffolds allowed sufficient transport of nutrients, oxygen, and wastes for growth of neurons,^[^ ^14^, ^15^ ^]^ which then promoted the improvements in motor function and tissue regeneration observed in this group. These results can be contrasted with the relatively ineffective results as obtained using control gelatin scaffolds without voids as reported previously.^[^ ^38^, ^44^, ^45^ ^]^ Nissl staining revealed large lesion volumes and disordered structures in the SCI and SCI + control GS groups versus that in the SCI + 3D GMS group, which showed significant reductions in cavities (Figure 4b). Moreover, scanning electron microscopy showed that lesion sites in SCI samples were nearly empty, and those in the SCI + control GS group were slightly reduced. However, the number of cavities within the SCI + 3D GMS group was significant reduced or absent, and those present were filled with solid tissue that was closely connected with the host spinal cord (Figure 4c). These results provided further evidence that 3D GM scaffolds improved the structural components of tissue regeneration and recovery after SCI.

Cell apoptosis was assessed at 28 d postinjury with transferase‐mediated dUTP‐biotin nick end labeling (TUNEL) staining. There were no statistically significant differences in the number of TUNEL‐positive cells at the injury sites among the three groups. These findings demonstrated the exceptional biocompatibility of the implanted materials (Figure S9a,b, Supporting Information).

### 2.4 3D GM Scaffolds Attenuate Immune Responses Following Spinal Cord Hemisection

**Figure 5:**
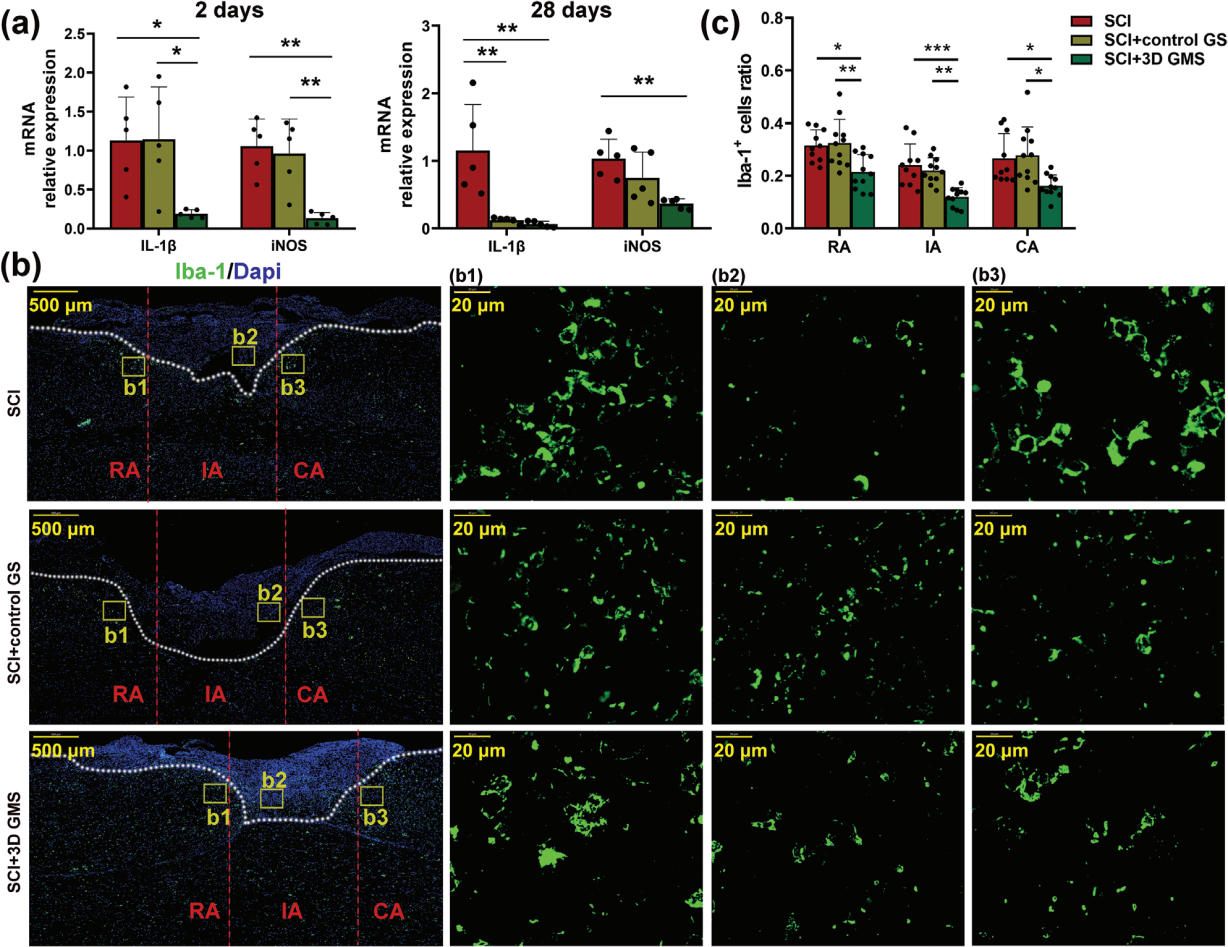
3D GM scaffolds attenuated microglia activation after SCI. a) qRT‐PCR assay of IL‐1*β* and iNOS mRNA expression at 2 and 28 d following injury and respective statistical analysis. *N*  =  5 rats per group. b) Representative images of Iba‐1 (green) immunohistochemical staining at 28 d after injury. The cavity boundary was indicated by the dashed lines. All cell nuclei were counterstained with Dapi (blue). Scale bar = 500 µm. b1–b3) Magnification of yellow frames (lesion core) in (b). Scale bar = 20 µm. c) Quantitative analysis of the ratio of Iba‐1^+^ microglia/macrophages in the area of lesioned areas (IA), rostral areas (RA) and caudal areas (CA). *N* = 9 in SCI group, *N* = 11 in SCI + control GS group, *N* = 11 in SCI + 3D GMS group; Values represent the mean ± SD, *p < 0.05, **p < 0.01, ***p < 0.001 according to ANOVA.

Secondary injuries, such as inflammatory responses and scar formation, can be problematic with tissue‐engineered transplantations, which show a strong association with their scaffolds and/or degradation products.^[^ ^49^ ^]^ As an food and drug administration (FDA)‐approved biomaterial for clinical use, gelatin has been shown to exhibit an excellent degree of biocompatibility.^[^ ^34^, ^50^, ^51^, ^52^ ^]^ To examine the effects of 3D GM scaffolds on the modulation of inflammatory responses, mRNA levels of inducible nitric oxide synthase (iNOS) and interleukin (IL)‐1*β* were measured with quantitative real‐time polymerase chain reaction (qRT‐PCR). When assessed at 2 d post‐SCI, the 3D GM scaffold group showed significantly reduced levels of IL‐1*β* [*F*(2,12) = 5.922, *p* < 0.05] and iNOS [*F*(2,12) = 11.938, *p* < 0.01] as compared with that obtained in both the SCI (IL‐1*β*: post hoc *p* < 0.05; iNOS: post hoc *p* < 0.01) and control GS (IL‐1*β*: post hoc *p* < 0.05; iNOS: post hoc *p* < 0.01) groups (**Figure** **5**a). 3D GM scaffolds significantly reduced the levels of IL‐1*β* [*F*(2,12) = 10.700, *p* < 0.01] and iNOS [*F*(2,12) = 20.399, *p* < 0.001] as compared with that obtained in the SCI group (IL‐1*β*: post hoc *p* < 0.05; iNOS: post hoc *p* < 0.001) and control GS (IL‐1*β*: post hoc *p* < 0.01; iNOS: post hoc *p* < 0.01) groups at 7 d (Figure S10, Supporting Information). Moreover, 3D GM scaffolds significantly reduced the levels of IL‐1*β* [*F*(2,12) = 11.88, *p* < 0.01; post hoc *p* < 0.01) and iNOS [*F*(2,12) = 7.211, *p* < 0.01; post hoc *p* < 0.01] as compared with that obtained in the SCI group at 28 d following SCI (Figure 5a).

Immunofluorescence was then used to identify any inflammatory responses present at these injury sites. We found that Iba‐1^+^ cells accumulated at the SCI site (Figure 5b,b2) and were also detected in rostral and caudal areas (CA) near injury sites (Figure 5b,b1,b3) at 28 d following injury. Most Iba‐1^+^ cells within SCI animals were characterized by a rounded amoeboid‐like appearance (microglia in an activated state). However, most Iba‐1 labeled cells in SCI + 3D GMS animals were characterized by small cellular bodies with ramified processes (microglia in a resting state). Iba‐1 immunoreactivity was quantified in three regions: rostral area, injury site and caudal area (Figure 5c). The number of spinal cord Iba‐1^+^ cells was significantly lower in the SCI + 3D GMS group (rostral area: *F*(2,29) = 7.249, *p* < 0.01; injury site: *F*(2,29) = 13.716, *p* < 0.001; caudal area: *F*(2,29) = 5.98, *p* < 0.01) as compared with the SCI group (rostral area: post hoc *p* < 0.05; injury site: post hoc *p* < 0.001; caudal area: post hoc *p* < 0.05) and SCI + control GS group (rostral area: post hoc *p* < 0.01; injury site: post hoc *p* < 0.01; caudal area: post hoc *p* < 0.05) (Figure 5c). Results from Western blot also showed that Iba‐1 was significantly downregulated in the injury core area of the SCI + 3D GMS group [*F*(2,12) = 40.965, *p* < 0.001] as compared with the SCI (post hoc *p* < 0.001) and SCI + control GS group (post hoc *p* < 0.001) (Figure S11, Supporting Information).

### 2.5 3D GM Scaffolds Attenuate Glial Scar Formation Following Spinal Cord Hemisection

**Figure 6:**
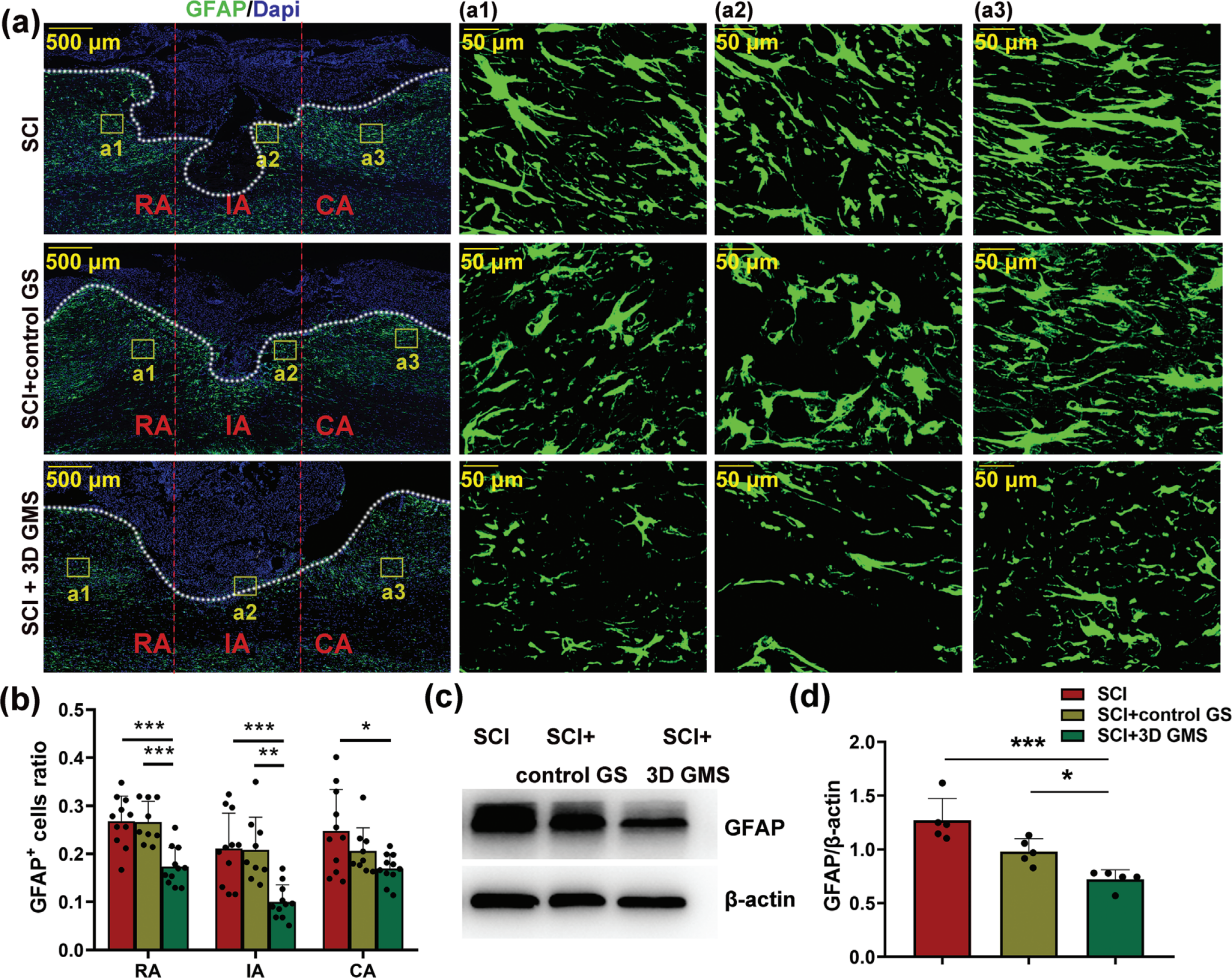
3D GM scaffolds attenuated glial scar formation after SCI. a) Representative images of GFAP (green) immunohistochemical staining at 28 d after injury. The cavity boundary was indicated by the dashed lines. All cell nuclei were counterstained with Dapi (blue). Scale bar = 500 µm. a1–a3) Magnification of yellow frames (lesion core) in (a). Scale bar = 50 µm. b) Quantitative analysis of the ratio of GFAP^+^ cells in the area of lesioned areas (IA), rostral areas (RA), and caudal areas (CA). *N* = 11 in SCI group, *N* = 9 in SCI + control GS group, *N* = 11 in SCI + 3D GMS group. c) Western blot assay of GFAP protein expression in three groups. d) Respective statistical analysis of GFAP. *N*  =  5 rats per group. Values represent the mean ± SD, *p < 0.05, **p < 0.01, ***p < 0.001 according to ANOVA.

As increased expression levels of glial fibrillary acidic protein (GFAP) may reflect the formation of glial scars, we examined the expression of GFAP in spinal cord tissue at 28 d after SCI with use of immunofluorescent staining. After SCI, GFAP^+^ cells were markedly increased at the broken areas of the injury site, seemingly forming an astrocytic wall (**Figure** **6**a,a1,a3), however very few GFAP^+^ cells were present at the injury site (Figure 6a, 2). In the SCI + 3D GMS group, numerous GFAP^+^ cells were present at the border of the injury site, but the immunoreactivity was noticeably less intense (Figure 6a) when compared with that in the SCI and SCI + control GS groups. Moreover, the SCI + 3D GMS group had a lower percent of GFAP immunoreactivity in the rostral area [*F*(2,28) = 14.931, *p* < 0.001], injury sites [*F*(2,28) = 11.572, *p* < 0.001] and caudal area [*F*(2,28) = 4.555, *p* < 0.05] as compared with the SCI group (rostral: post hoc *p* < 0.001; injury sites: post hoc *p* < 0.001; and caudal: post hoc *p* < 0.05) and SCI + control GS group (rostral: post hoc *p* < 0.001; injury sites: post hoc *p* < 0.01; and caudal: post hoc *p* > 0.05) (Figure 6b).

Results from Western blot also showed that GFAP was significantly downregulated in the injury core area of the SCI + 3D GMS group [*F*(2,12) = 17.686, *p* < 0.001] as compared with the SCI (post hoc *p* < 0.001) and SCI + control GS (post hoc *p* < 0.05) groups (Figure 6c,d). These results demonstrate that 3D GM scaffolds reduced the formation of glial scar after SCI. When combining these results with those obtained as described above, we conclude that a potential therapeutic mechanism of 3D GM scaffold implantation can be mainly attributable to the natural gelatin material as the degradation product of collagen. Such circumstances then have the capacity to produce a suitable anti‐inflammatory and trouble‐free migratory microenvironment for nerve regeneration under conditions of relatively low antigenicity.

### 2.6 3D GM Scaffolds Promote the Regeneration of Nerve Fibers Following Spinal Cord Hemisection

**Figure 7:**
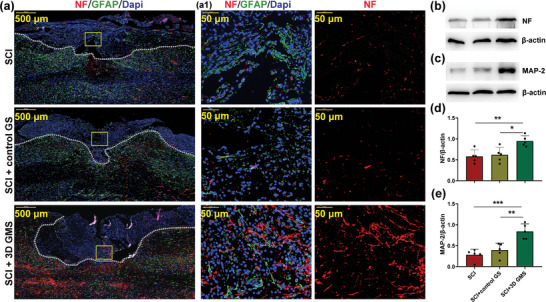
3D GM scaffolds boost regeneration of nerve fibers at 28 d after SCI. a) Representative images of NF (red) and GFAP (green) immunohistochemical staining at 28 d after injury in spinal cord lesion areas. All cell nuclei were counterstained with Dapi (blue). The cavity boundary was indicated by the dashed lines. Scale bar = 500 µm. a1) Magnification of yellow frames (lesion core) in (a). Scale bar = 50 µm. b) Western blot assay of NF protein expression in three groups. c) Western blot assay of MAP‐2 protein expression in three groups and respective statistical analysis. d) Quantitative analysis of NF levels in three groups. *N*  =  5 rats per group. e) Quantitative analysis of MAP‐2 levels in three groups. *N*  =  5 rats per group. Values represent the mean ± SD, *p < 0.05, **p < 0.01, ***p < 0.001 according to ANOVA.

Axonal regeneration of the neuron throughout the injury site is critical for effective SCI repair. Accordingly, an immunostaining analysis of the 200 kDa subunit of neurofilament‐L (NF) was employed to evaluate neuronal and axonal damage, as described previously.^[^ ^39^ ^]^ In comparison with that observed in the SCI group, a significant increase in NF labeling adjacent to the injury site was observed in the SCI + 3D GMS group, suggesting that the 3D GM scaffolds were exerting a continuous, protective effect in these neurons (**Figure** **7**a). Western blot results showed that NF expression in the SCI + 3D GMS group was significantly increased [*F*(2,12) = 8.481, *p* < 0.01] as compared with that in both the SCI (post hoc *p <* 0.01) and control GS (post hoc *p* < 0.05) groups at 28 d following SCI (Figure 7b,d).

Microtubule associated protein 2 (MAP‐2), a specific structural protein associated with neuronal growth, was assessed and quantified with Western blot. We found that MAP‐2 expression was significantly increased in the SCI + 3D GMS group [*F*(2,12) = 16.978, *p* < 0.001] as compared with that in both the SCI (post hoc *p* < 0.001) and control GS (post hoc *p* < 0.01) groups (Figure 7c,e). Moreover, results from immunofluorescent staining demonstrated that a significant increase in MAP‐2 labeling was present adjacent to the injury site in the SCI + 3D GMS group as compared with the SCI and SCI + control GS groups (Figure S12, Supporting Information). These results indicate that these 3D GM scaffolds not only exerted positive effects upon supporting the viability of remaining axons but also in encouraging the growth of new axons.

### 2.7 Therapeutic Effects of 3D GM Scaffolds Are Linked with the Activation of AKT and ERK Pathways

**Figure 8:**
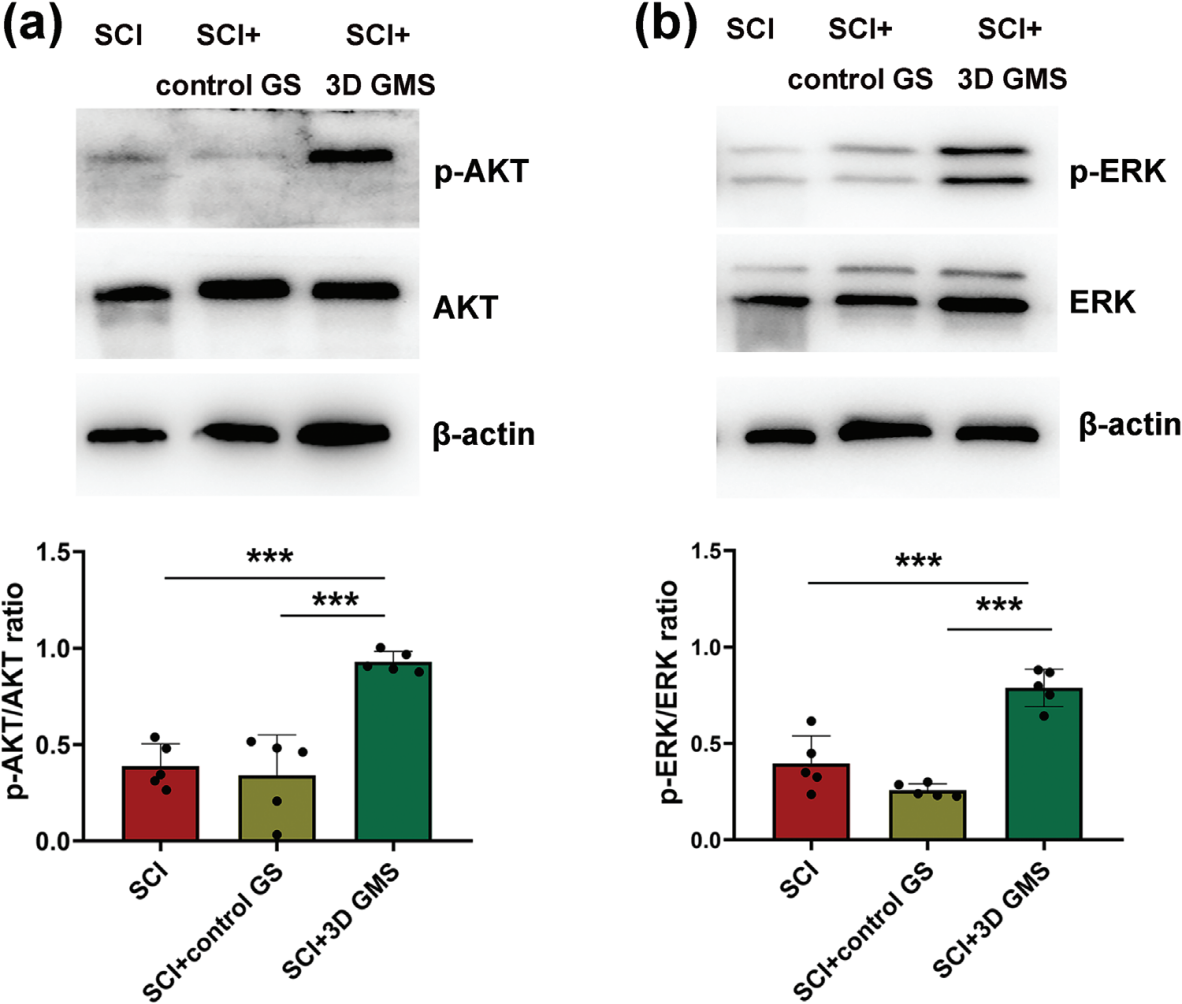
Therapeutic effects of 3D GM scaffolds are linked to activate AKT and ERK pathways. a) Western blot assay of p‐AKT and AKT expression in three groups and respective statistical analysis. b) Western blot assay of p‐ERK and ERK protein expression in three groups, and respective statistical analysis. *N*  =  5 rats per group. Values represent the mean ± SD, ***p < 0.001 according to ANOVA.

Protein kinase B (AKT) and extracellular signal‐regulated kinase (ERK) pathways play an important role in neuronal survival, axonal outgrowth and synaptic plasticity.^[^ ^8^, ^53^, ^54^, ^55^, ^56^ ^]^ High expression levels of AKT within the nervous system are related to neuroprotective effects,^[^ ^54^ ^]^ while increased ERK phosphorylation is involved in preconditioning as well as in some natural products associated with neuroprotection.^[^ ^57^, ^58^ ^]^ To explore whether the beneficial effects of these 3D GM scaffolds in promoting SCI recovery were related to AKT and ERK activation, we first measured expression levels of p‐AKT, AKT, p‐ERK, and ERK in each group at 28 d following SCI by Western blot. Our results revealed that there were significant increases in the ratios of p‐AKT/AKT [*F*(2,12) = 26.367, *p* < 0.001] and p‐ERK/ERK [*F*(2,12) = 36.285, *p* < 0.001] in the SCI + 3D GMS group as compared with that in the SCI (p‐AKT/AKT: post hoc *p* < 0.001; p‐ERK/ERK: post hoc *p* < 0.001) and SCI + control GS (p‐AKT/AKT: post hoc *p* < 0.001; p‐ERK/ERK: post hoc *p* < 0.001) groups (**Figure** **8**). These results imply that activation of the AKT and ERK pathways plays an important role in the neuronal repair as produced with 3D GMS scaffolds.

## 3 Conclusions

The 3D GM scaffolds used in this study exhibited well‐connected 3D porous structures, appropriate void sizes, exceptional biocompatibility and plasticity, suitable porosity and degradation, as well as low preparation costs and a convenience of production. Through our comprehensive analyses we were able to demonstrate that these 3D GM scaffolds effectively bridged injury gaps, promoted tissue regeneration, established nerve connections and signal transductions, mitigated the inflammatory microenvironment and reduced the formation of glial scars. Taken together, our results demonstrate that 3D GM scaffolds provide a novel and effective mean of bridging injury gaps in SCI. Therefore, this procedure can serve as an effective approach for use in promoting the regeneration and reconstruction of nerves to improve the recovery of neuronal function following SCI.

## 4 Experimental Section

### Ethics

All experimental procedures in this study were approved by the Animal Ethics and Welfare Committee of Shandong University (approval No. ECSBMSSDU 2018‐2‐059). All animals were permitted free access to food and water and were maintained in a pathogen‐free environment with a temperature of 22–24 °C, 60% relative humidity, and a 12 h light‐dark cycle in the Laboratory Animal Center of Shandong University.

### Materials

Gelatin (type B, from porcine skin) was purchased from Sigma‐Aldrich, methanol (anhydrous) from Macklin, toluene from Sinopharm and Span‐80 from Macklin. All chemicals were used as received. PVC tubes (inner diameter: 0.9 mm) were purchased from Thermo Scientific, glass microcapillary tubes (inner diameter: 0.5 mm) from Aceglass and needles (26 g) from Shihva. The epoxy adhesive was purchased from Devtube. All water used in the experiments was obtained following filtration through Millipore cartridges (Epure, Dubuque, IA).

### Preparation and Characterization of Microspheres Using the Fluidic Device

The microfluidic device consisted of a PVC tube, a microcapillary tube, and a 26 g needle. The key to control the consistency of scaffolds in each preparation is to use homogeneous gelatin microspheres. In order to obtain homogeneous gelatin microspheres, a microfluidic device was constructed by inserting the needle and microcapillary tube into the PVC tube, which was then fixed with epoxy adhesive based on the previous study (Figure 1a and Figure S1, Supporting Information). Discontinuous and continuous phases were introduced using syringe pumps (LSP02‐2B, Longer) with independently adjusted flow rates. By constantly adjusting the parameters (e.g., the velocity and density of discontinuous phase, inner diameter of capillary tube, and velocity of the continuous phase) to obtain the optimal capillary number and Weber number, which described the combined effects of viscous shear force and inertial force of internal fluid with respect to surface tension, and finally to obtain the homogeneous O/W droplets at the end of the needle. Based on a large number of experimental explorations, toluene (containing 2% span‐80) and gelatin aqueous solutions (10 wt%) were selected as continuous phase and discontinuous phase, respectively, and the flow rates for the discontinuous and continuous phases were maintained at 0.05 and 2.0 mL min^−1^, respectively. Droplets formed at the tip of the needle flowed along the glass capillary tube into a 1 L tall beaker containing 900 mL of the collection phase (span‐80), which was then gently stirred overnight to allow solvent evaporation. The syringe filled with gelatin solution was heated to 60 °C with use of heating tape to prevent gelation. Finally, precise control over particle size of gelatin microspheres was realized. SEM (S‐4800, Hitachi, Japan) was used to assess the morphology of microspheres. The average diameter was calculated from these SEM images by Image J software as accomplished by measuring the diameters of over 100 randomly selected particles.

### Fabrication of 3D GM Scaffolds

An appropriate number of gelatin microspheres was dispersed in 5 mL methanol and placed in a 50 mL centrifuge tube (Nest). After the microbeads had completely settled to the bottom of the tube, the centrifuge tube was gently tapped for 1 min to facilitate the formation of a ccp lattice. The resultant ccp lattice was placed in an oven and preheated to 80 °C for 15 min to produce necking (partial fusion) between adjacent microbeads. The pellet was frozen at −80 °C for 30 min and then lyophilized in a freeze‐dryer (Biocool) overnight. Finally, ccp lattice gelatin microspheres were obtained. The morphologies, compositions, and mechanical characterizations of 3D GM scaffolds were assessed under SEM, FTIR (NEXUS 670, Thermo Nicolet) and an atomic force microscopy (Dimension icon, Bruker, Germany).

### Fabrication of Control Gelatin Scaffolds

Control gelatin scaffolds were also produced from the aqueous solution of 10 wt% gelatin without the microfluidic molding process. Gelatin (1 g) was added to 9 mL deionized water to prepare the 10 wt% gelatin aqueous solution. The solution was heated to 80 °C and then slowly poured into a 1.5 mL centrifuge tube to prevent any air bubble production. Control gelatin scaffolds were gradually solidified into a structure without voids as achieved with a decreasing temperature and lyophilized overnight. Morphologies of control gelatin scaffolds were assessed using SEM.

### Spinal Cord Hemisection Model and Surgical Procedures

Six‐week‐old male Sprague‐Dawley (SD) rats were used in these experiments. Spinal cord hemitransections were performed after 3 d of acclimatization to laboratory conditions and a half day of food and water deprivation (Figure S6, Supporting Information). Surgeries were performed while the rats were under anesthesia (10% chloral hydrate, ip). After shaving and disinfecting the area, skin, fascia, and muscles were separated successively to expose the lamina and a T10 thoracic laminectomy was performed. The spinal cord was exposed and in an orientation perpendicular to the spinal cord a spinal cord segment of ≈2 mm to the right of T10 was removed with a microscalpel. The tissue fluid was aspirated and pre‐prepared gelatin microspheres (with or without 3D GM scaffolds) were implanted after the remaining fibers were fully removed. A scaffold was used to plug the defect area of the spinal cord, the wound was sutured and disinfected (Figure S7a, Supporting Information). Observation of a tail spasm and tremor of the right lower limb after paralysis indicated that the spinal cord hemisection was successful. The animals were treated with penicillin once a day for three consecutive days after the surgery and their bladders was manually compressed to promote the recovery of spontaneous urination. This SCI rat model was randomly divided into three groups with 25 rats per group: 1) SCI only, 2) SCI + control gelatin scaffolds (SCI + control GS), and 3) SCI + 3D GMS.

### Functional Studies

To evaluate locomotor activity, the rats were placed in a 125 × 125 cm^2^ open arena and observed for 4 min. They were scored according to the BBB locomotor rating scale.^[^ ^59^ ^]^ BBB scores were recorded on days 1, 3, 7, 14, 21, and 28 after surgery. All evaluations were performed at the same time to avoid any influence of the circadian rhythm on experimental results. Observers were blind as to the grouping and treatment of rats.

### Footprint Analysis of Gait

A gait analysis test was performed to provide an accurate evaluation of their limb coordination.^[^ ^60^ ^]^ Gait analysis tests were conducted on days 28 after surgery. For these tests, the forelimbs were colored with red ink and hind limbs with black ink and the rats were placed at one end of a glass test tank (1 m × 12 cm) containing white blotting paper. When they reached the end of the tank, the rats were removed. The start and end positions for each test were fixed, and each rat was tested three times. Gait analysis was evaluated as based on stride length (cm), defined as the distance between successive placements of the same paw.^[^ ^61^ ^]^ Six continuous steps were randomly selected and the average stride length value was calculated. Representative results of gait blotting experiments were selected and scanned into images.

### MEP

MEP were recorded at 24 h before SCI and on days 1, 14, and 28 post‐SCI. MEP was elicited from a stimulating electrode implanted at 2.5 mm anterior to bregma, 2.5 mm lateral to the midline, and at a depth of 1 mm for insertion into the M1 primary motor area. An indwelling negative electrode was inserted into the skin of the head. Positive and negative recording electrodes were inserted into the gastrocnemius muscle, with a distance of 1 cm between recording electrodes.^[^ ^62^, ^63^ ^]^ Both stimulation and the recording electrodes were connected to a Biological Information Acquisition and Analysis System BL‐420F. Amplitudes of MEP were defined as the maximum distance between positive and negative peaks. Stimulation parameters were set at a frequency of 10 Hz, a delay of 100 ms, a wave width of 1 ms, a wave interval of 10 ms, and a stimulation intensity of 5 mA. MEP was recorded after the potential had stabilized.

### Tissue Preparation, Histological Staining, and Immunofluorescent Labeling

At 28 d post‐SCI, a portion of the injury core area of the spinal cord (3 mm) was removed and fixed in 4% paraformaldehyde for >24 h. The middle region consisted of the 2 mm core area of the lesion, while the 0.5 mm tissue areas on both sides were labeled as the rostral and caudal sides. Spinal cord tissue was dehydrated in increasing concentrations of ethanol, embedded in paraffin, cooled and solidified on a freezing table at −20 °C, and then sectioned (4 µm) along the longitudinal axis. The slices were subjected to antigen retrieval and goat serum treatment, and incubated overnight at 4 °C with the following primary antibodies: astrocyte marker GFAP (Mouse monoclonal, Proteintech, 1:500), mature neuron marker MAP‐2 (Rabbit polyclonal, Abcam, 1:500), axon marker NF (Rabbit monoclonal, Cell Signaling Technology, 1:500) and microglia marker Iba‐1 (Mouse monoclonal, Abcam, 1:500). After washing with phosphate buffered saline (PBS), the secondary antibodies (Jackson ImmunoResearch Alexa Fluor488‐conjugated AffiniPure Goat Anti‐mouse IgG (H + L) and Alexa Fluor594‐conjugated AffiniPure Goat Anti‐Rabbit IgG (H + L)) were incubated for 30 min. Fluorescent microscopy (OLYMPUS‐BX51) and the Magna Fire SP system were used to analyze the images. GFAP and Iba‐1 immunofluorescent staining were used to identify glial scars and microglial cells which were expressed as the percent of positive cells to the total number of cells.

### Nissl Staining of Spinal Cord Slices

Spinal cord slices were deparaffinized in xylene, washed with water, and immersed in toluidine blue staining solution for 2–5 min. The slices were then differentiated with 0.1% glacial acetic acid, which controlled for differentiation when viewed under the microscope. Slices were then washed with water to stop the reaction. After drying in the oven, slices were soaked in xylene for 10 min, and sealed with neutral gum with images collected under microscopy (Zeiss Axio Imager 2) for subsequent analysis. Regions of traumatic injury were identified as based on severe tissue destruction and/or staining loss.

### Terminal Deoxynucleotidyl TUNEL Staining

TUNEL staining was performed according to instructions of the TUNEL kit (Servicebio Fluorescein (FITC) TUNEL Cell apoptosis Detection Kit). Briefly, sections were deparaffinized and hydrated followed by incubation in 20 µg mL^−1^ proteinase k working solution at 37 °C for 20 min, washed with PBS, and then treated with rupture fluid at room temperature for 20 min. Equilibration Buffer (50 µL) was added dropwise and sections were incubated at room temperature for 20 min followed by incubation with a TdT enzyme reaction solution at 37 °C for 1–2 h. Streptavidin‐tritc marker solution was then added to each sample and incubated at 37 °C for 30 min. Finally, six fields of view (40 ×) were randomly searched in the core area of the lesion in each section and the proportion of TUNEL‐positive cells was expressed as the percent of the total cells counted.

### Scanning Electron Microscopy

Fresh spinal cord tissue was removed from the injury core area, gently rinsed with PBS to remove blood stains, and fixed in the electron microscopy fixation solution (2.5% glutaraldehyde, 100 mm phosphate, pH 7.0–7.5). Fixed samples were rinsed three times with 0.1 m phosphate buffer (pH = 7.4) for 15 min, each followed by fixation with 1% osmium acid at room temperature in the dark for 1–2 h. Samples were rinsed again and dehydrated in varying ethanol gradient concentrations for 15 min. Finally, the sample was dried in a critical point drying instrument (Quorum K850), adhered to the conductive carbon film double‐sided tape, and placed on the sample stage of an ion sputtering instrument (HITACHI MC1000) for a 30 s spraying of gold. Samples were then collected and observed under SEM.

### Western Blot Analysis

Fresh tissue samples from the core injury area of the spinal cord were removed, lysed in radio immunoprecipitation assay (RIPA) lysis buffer containing phenylmethylsulfonyl fluoride (PMSF) and protease inhibitor (PI) for 10–30 min, and then centrifuged at 12 000 × *g* for 10 min at 4 °C after lysis. The supernatant was aspirated and the bicinchoninic acid (BCA)detection kit was used to quantify proteins, with sodium dodecyl sulfate ‐ polyacrylamide gel electrophoresis (SDS‐PAGE) gels used to concentrate and separate proteins at different constant voltages. Proteins were then transferred to polyvinylidene fluoride (PVDF) membranes under a constant current and blocked with a Tris Buffered Saline with Tween20 (TBST) solution containing 5% skim milk for 1 h at room temperature. The PVDF membrane was then incubated with primary antibodies overnight at 4 °C: *β*‐actin (Mouse monoclonal, ZSGB‐BIO, 1:1000), GFAP (Mouse monoclonal, Proteintech, 1:1000), MAP‐2 (Rabbit polyclonal, Abcam, 1:1000), axon marker NF (Rabbit monoclonal, Cell Signaling Technology, 1:1000), Iba‐1 (Mouse monoclonal, Abcam, 1:500), AKT (Rabbit polyclonal, Cell Signaling Technology, 1:1000), p‐AKT (Rabbit polyclonal, Cell Signaling Technology, 1:1000), ERK (Rabbit polyclonal, Cell Signaling Technology, 1:1000), and p‐ERK (Rabbit polyclonal, Cell Signaling Technology, 1:1000). After three washes with TBST solution, PVDF membranes were incubated with the secondary antibody for 1 h. With use of the Tanon system, images were developed with enhanced chemiluminescence (ECL) developer solution and gray values calculated by image J software to detect protein expression.

### Reverse Transcriptase qRT‐PCR

Tissues from rat spinal cord injury regions (the 2 mm lesioned areas and 0.5 mm rostral and caudal areas) were freshly extracted for determination of total RNA with use of the Ultrapure RNA kit (CWBIO, China). Briefly, tissues were fully lysed using TRIzol Lysis Solution (Beyotime, China) and mixed with chloroform in a ratio of 1:1 followed by centrifugation at 12 000 rpm for 10 min. The colorless and clear supernatant obtained was mixed with an equal volume of 70% absolute ethanol and washed several times followed by a 12 000 rpm centrifugation with the cleaning solution in the adsorption column to obtain purified total RNA. Complementary DNA was then synthesized using the ReverTra Ace Qpcr RT Kit (TOYOBO, Tokyo, Japan). According to the instructions provided, quantitative real‐time PCR was performed using synergy brands (SYBR) green mix (TOYOBO, Tokyo, Japan) with the Bio‐rad IQ5 Real Time PCR System (Bio‐Rad, USA). Primer sequences used for qPCR amplification were

IL‐1*β*: 5′‐GCAACTGTTCCTGAACTCAACT‐3′ (forward)
5′‐ATCTTTTGGGGTCCGTCAACT‐3′ (reverse).
iNOS: 5′‐CTCCTTCAAAGAGGCAAAAATA‐3′ (forward)
5′‐CACTTCCTCCAGGATGTTGT‐3′ (reverse).
*β*‐actin: 5′‐CTATTGGCAACGAGCGGTTCC ‐3′ (forward)
5′‐CAGCACTGTGTTGGCATAGAG‐3′ (reverse).

PCR amplification was performed with cycling conditions that included a 2 min initial denaturation at 94 °C, followed by 40 cycles of 94 °C for 15 s, 60 °C for 20 s, and 72 °C for 30 s. Target gene expressions were normalized to *β*‐actin (internal control) using the 2^−ΔΔCt^ method.

### Cell Culture

After isolation from bone marrow of SD rats, MSCs cultured Dulbecco's modified Eagle's medium (Gibco) supplemented with 10% fetal bovine serum (Gibco), and 10 mg mL^−1^ penicillin and 10 mg mL^−1^ streptomycin in 5% CO_2_ at 37 °C. Passages 3–4 MSCs were cultured in 96 well plates. The 3D GM scaffolds were strictly sterilized before using. The 3D GM scaffolds were taken out for open field observation and immunofluorescence staining after 48 h incubating with MSCs. MSCs were labeled with Dapi and homing cell adhesion molecule (CD44) (Rabbit polyclonal, Proteintech, 1:500). In addition, another 3D GM scaffolds cocultured with MSCs were transferred into in a new 96 well plate for open field observation and immunofluorescence staining after 72 h.

### Statistical Analysis

Data were expressed as mean ± standard deviations (SD). The SPSS 22.0 software (IBM, USA) program was used to perform the statistical analyses. Absolute values or data normalized to control conditions were presented, as described in the respective figures. A repeated measure two‐way analysis of variance (ANONA) was used for group comparisons of BBB performance over time, with “days” as the within‐subject factor and “groups” as the between‐subject factor. One‐way ANOVAs with Bonferroni's correction were used for multiple group comparisons at each time point and step. Data distributions of Western blot and qRT‐PCR were analyzed using one‐way ANOVA with Bonferroni's correction for multiple pairwise comparisons. A *p* < 0 .05 was required for results to be considered as statistically significant.

## Conflict of Interest

The authors declare no conflict of interest.

## Supporting information

Supporting Information

Supplemental Video 1

Supplemental Video 2

Supplemental Video 3

## Data Availability

The data that support the findings of this study are available from the corresponding author upon reasonable request.
