# Supplementary material for: 3D Gelatin Microsphere Scaffolds Promote Functional Recovery after Spinal Cord Hemisection in Rats
Source: Adv Sci (Weinh). 2022 Dec 1;10(3):2204528. doi: 10.1002/advs.202204528 (PMC9875663; doi:10.1002/advs.202204528)
Supplement: Supplementary file 1 — Supporting Information [file ADVS-10-2204528-s004.pdf]

ADVANCED SCIENCE Open Access

Supporting Information

for *Adv. Sci.*, DOI 10.1002/advs.202204528

3D Gelatin Microsphere Scaffolds Promote Functional Recovery after Spinal Cord Hemisection in Rats

*Hongfei Ke, Hongru Yang, Yijing Zhao, Tingting Li, Danqing Xin, Chengcheng Gai, Zige Jiang and Zhen Wang**

3D Gelatin Microsphere Scaffolds Promote Functional Recovery after Spinal Cord Hemisection in Rats

*Hongfei Ke, Hongru Yang, Yijing Zhao, Tingting Li, Danqing Xin, Chengcheng Gai, Zige Jiang, Zhen Wang**

Supporting Information

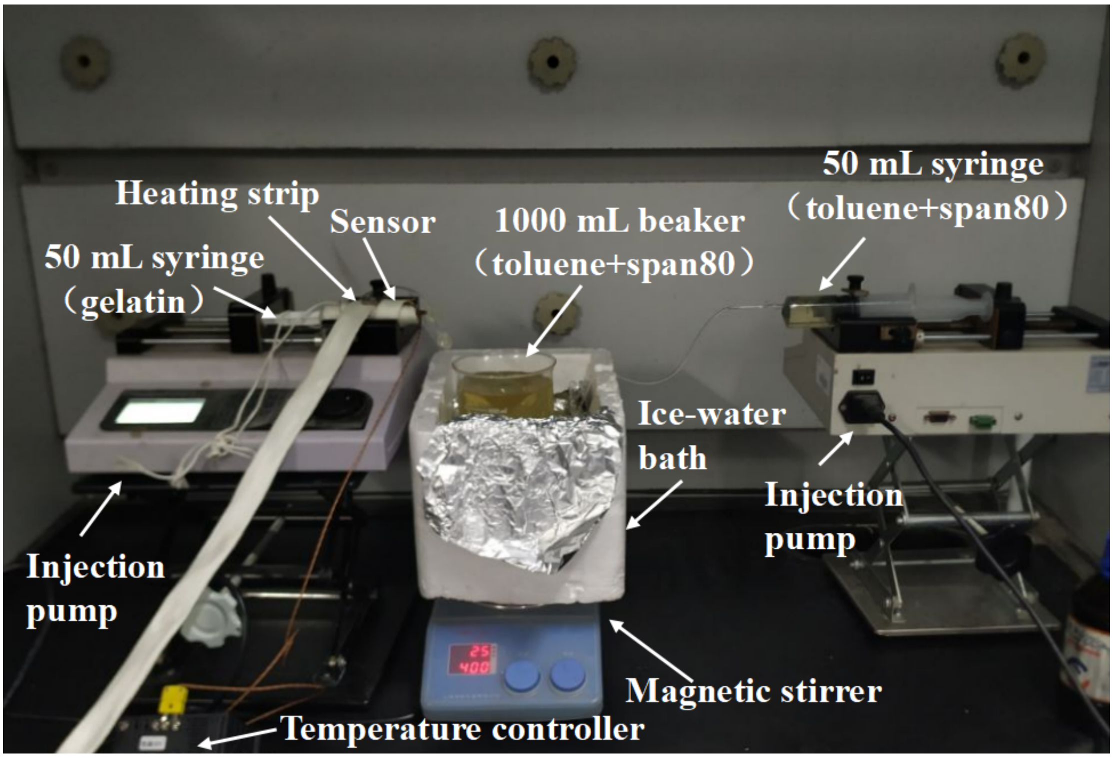

Figure S1. The assemblies of microfluidic device.

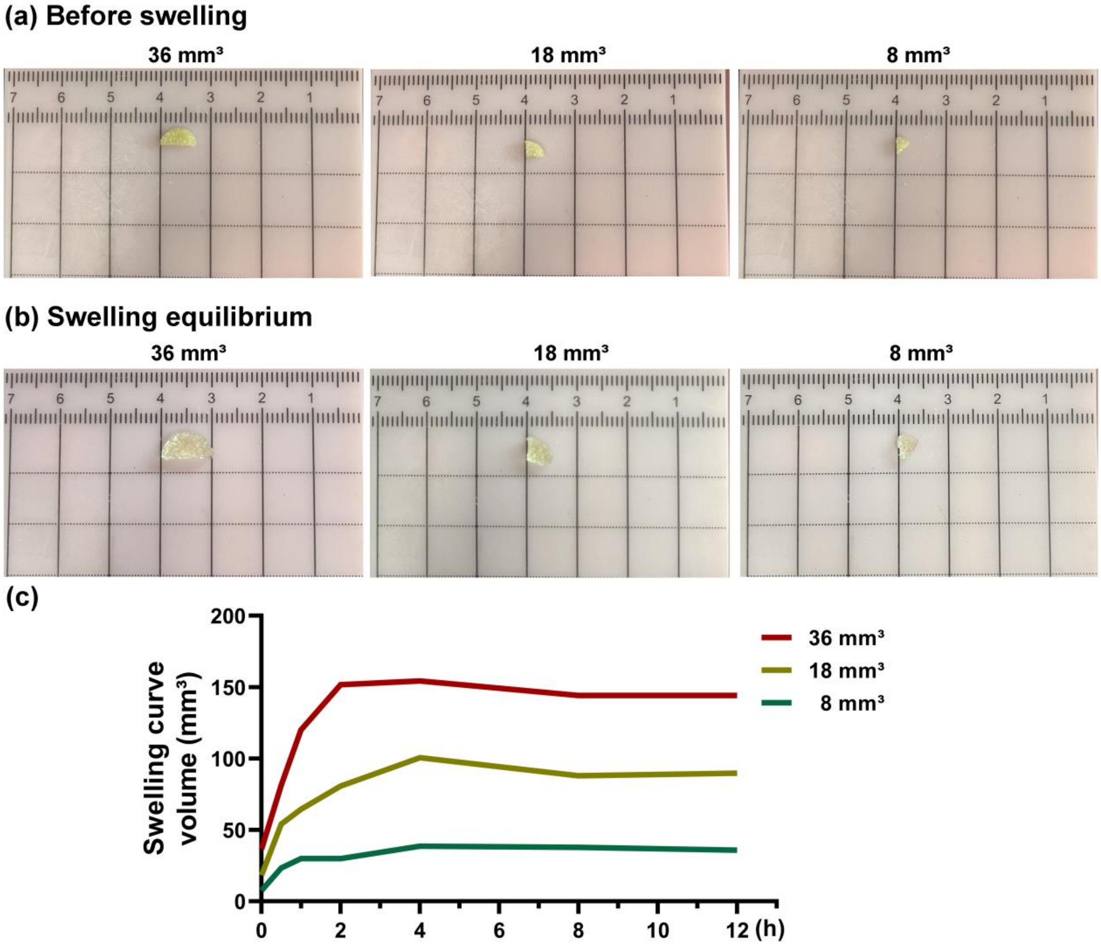

Figure S2. Swelling behavior of 3D gelatin microsphere scaffolds.

(a) Measurement diagram of 3D gelatin microsphere scaffolds with different volumes (36 mm^3, 18 mm^3 and 8 mm^3) before swelling. (b) Measurement diagram of 3D gelatin microsphere scaffolds with different volumes (36 mm^3, 18 mm^3 and 8 mm^3) after reaching swelling equilibrium. (c) Swelling curve of 3D gelatin microsphere scaffolds.

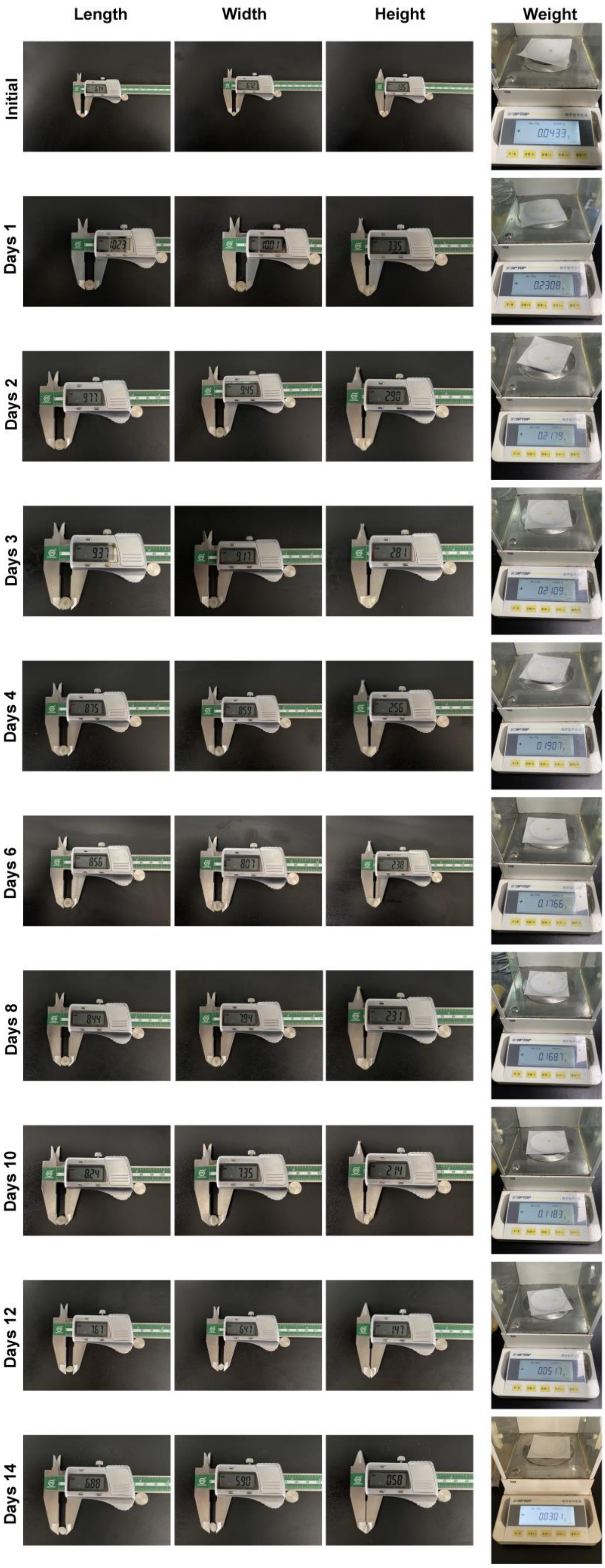
Length
Width
Height
Weight
Initial
Days 1
Days 2
Days 3
Days 4
Days 6
Days 8
Days 10
Days 12
Days 14

Figure S3. Representative images of the degradation of 3D gelatin microsphere scaffolds.

During the *in vitro* degradation experiment, accurately measured the length, width and height of the 3D gelatin microsphere scaffold at the 1, 2, 3, 4, 6, 8, 10, 12 and 14 days by the vernier caliper, and recorded weight with the electronic balance.

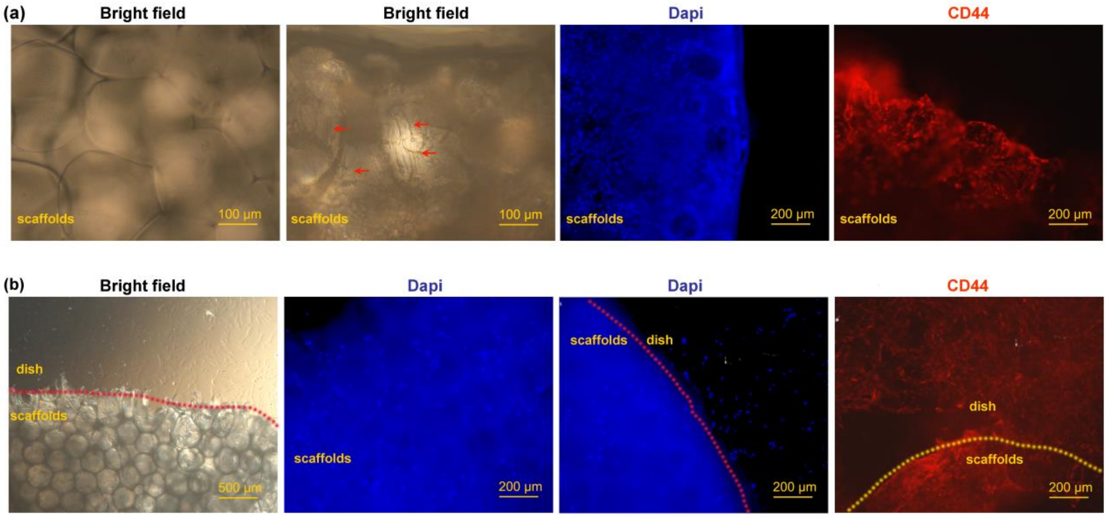

Figure S4. Mesenchymal stem cells (MSCs) can effectively adhere to and migrate on the 3D gelatin microsphere scaffolds

(a) Representative images of bright fields and fluorescent staining of 3D gelatin microsphere scaffolds with MSCs. The 3D gelatin microsphere scaffolds were cultured with/without MSCs suspension and culture medium, and cultured at 37 °C for 48 h. The 3D gelatin microsphere scaffolds were moved to a new culture dish for bright field and fluorescent staining observation. The image of left bright field shows 3D gelatin microsphere scaffolds cultured without MSCs. The image of right bright field show 3D gelatin microsphere scaffolds cultured with MSCs. Scale bar=100 μm in bright field. Scale bar=200 μm in Dapi and CD44 staining. Red arrows point MSCs.

Dapi (blue) and CD44 (red) were used to mark MSCs. (b) Representative images of bright fields and fluorescent staining of 3D gelatin microsphere scaffolds with MSCs in a new culture dish after 72 h. The boundary of 3D gelatin microsphere scaffolds and the culture dish was indicated by the dashed lines. Scale bar=500 μm in bright field. Scale bar=200 μm in Dapi and CD44 staining.

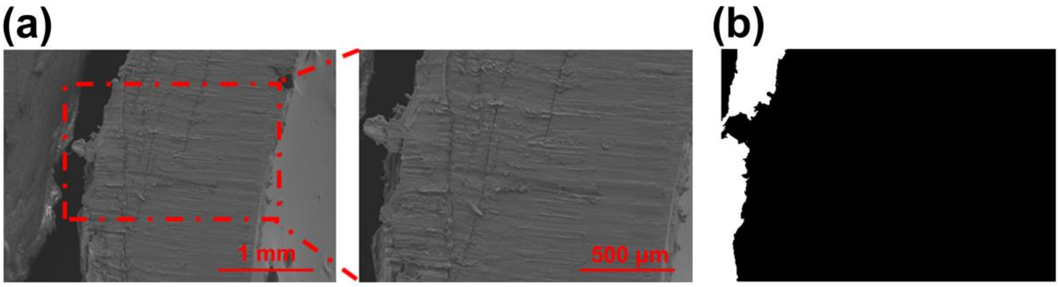

Figure S5. **The characterization of morphologies of control gelatin scaffolds.**

(a) The side-view SEM image of control gelatin scaffolds. Scale bar=1 mm in left image. Scale bar=500 μm in right image. (b) Diagram of microsphere area obtained through Image J transformation.

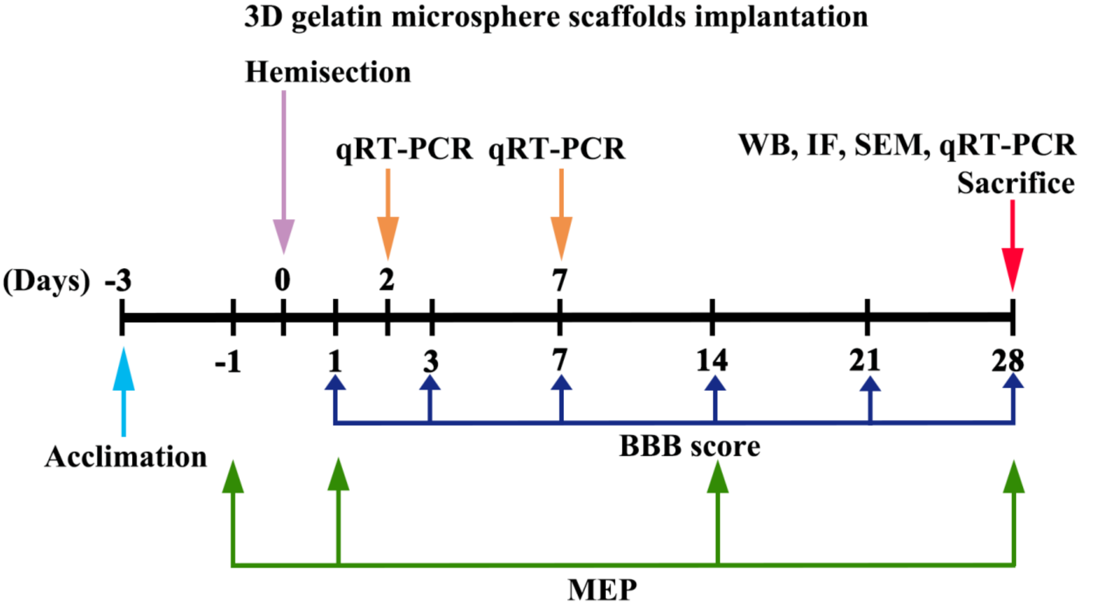

Figure S6. Schema for modeling method, behavior and molecular biology

experiment of rats treated with 3D gelatin microsphere scaffolds.

Spinal cord hemi-transections injuries were performed after 3 days of acclimatization to laboratory conditions and a half day of food and water deprivation. The MEP test was conducted 24 h before operation, 24 h, 7 days and 28 days after operation, respectively. BBB score was conducted on the 1, 3, 7, 14, 21 and 28 days after operation. The qRT-PCR was conducted on the 2, 7 and 28 days post-SCI, and WB, IF and SEM were performed on the 28 days post-SCI.

Abbreviation: qRT-PCR, Reverse transcriptase quantitative real-time polymerase chain reaction; **MEP**, Motor evoked potential; **BBB score**, Score of Basso, Beattie & Bresnahan locomotor rating scale; **WB**, Western blot; **IF**, Immunofluorescence; **SEM**, Scanning electron microscope.

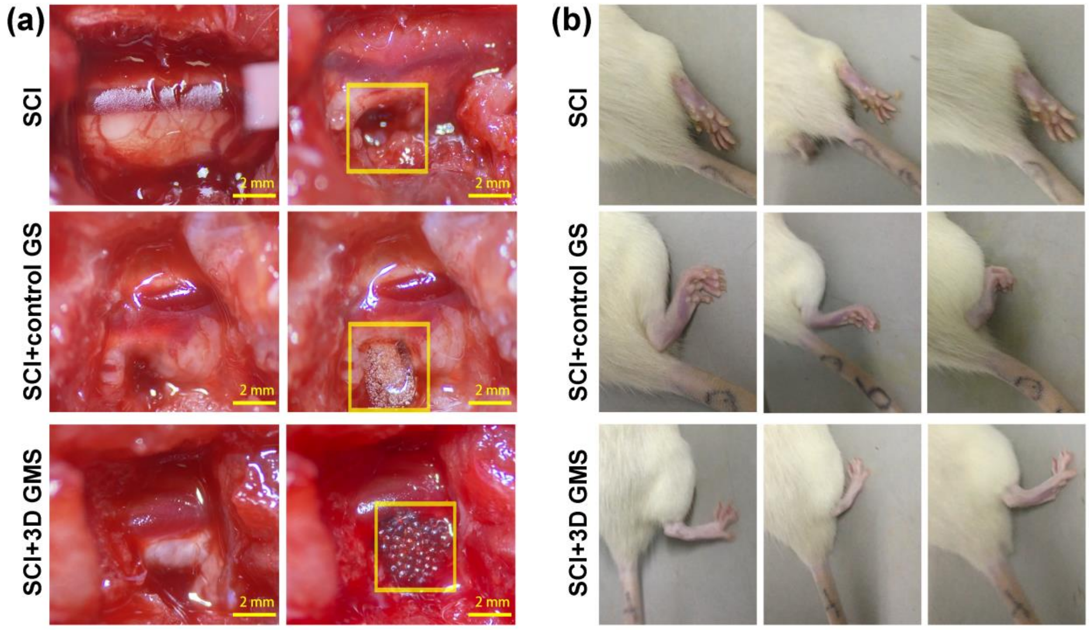

Figure S7. **Semi-transverse operation and right hind limb movement.**

(a) In the SCI group, the left figure showed spinal cord exposure, and in the right

figure showed yellow frames marked tissue defects after SCI. The left figure of the SCI+control GS group and SCI+3D GMS group shows tissue defect, and in the right figure showed yellow frames marked tissue defect implanted by scaffolds. Scale bar=2 mm. (b) Right hind limb movement during forward crawling in Days 14.

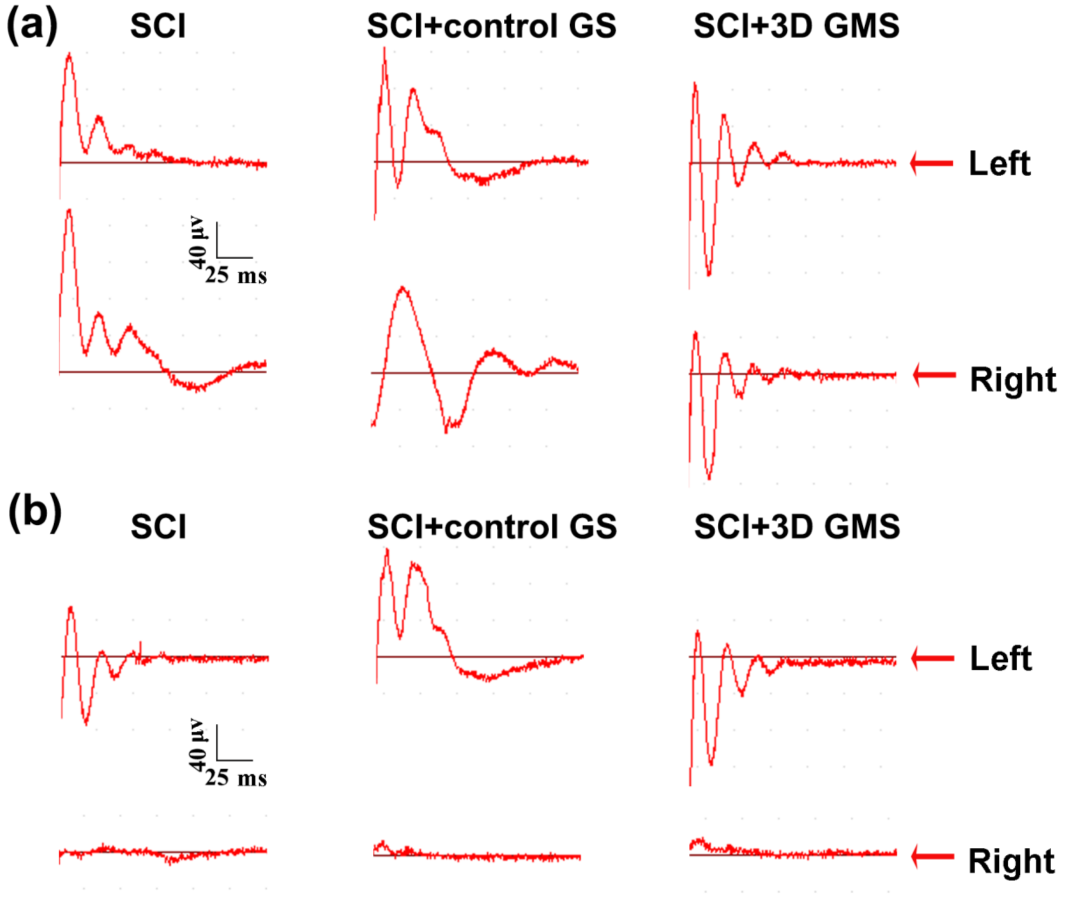

Figure S8. **Typical images of hindlimb motor evoked potentials.**

(a) Typical images of hindlimb motor evoked potentials in the three groups before surgery. (b) Typical images of hindlimb motor evoked potentials in the three groups after surgery.

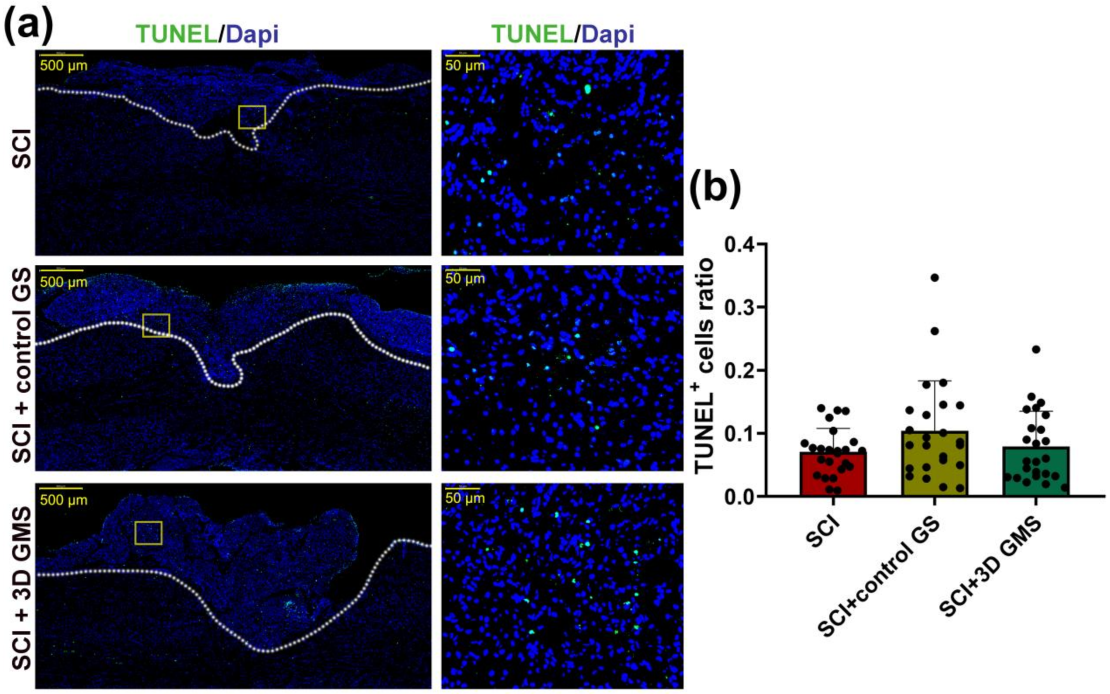

Figure S9. **TUNEL staining and analysis at 28 days after SCI.**

(a) Representative images of TUNEL (green) staining 28 days after SCI. All cell nuclei were counterstained with Dapi (blue). The cavity boundary was indicated by the dashed lines. Scale bar=500 µm. Magnification of yellow frames in the left figure. Scale bar=50 µm. (b) Quantitative analysis of the ratio of $TUNEL^+$ cells in lesion areas in each group, 6 random fields (20×) were selected for cell count in each section, N = 4 rats per group. Values represent the mean ± SD.

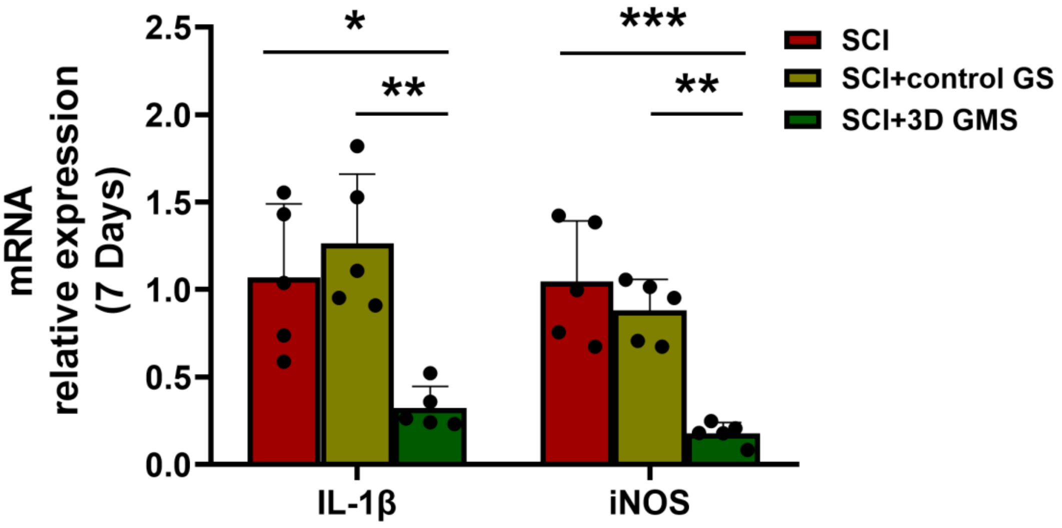

Figure S10. 3D GM scaffolds attenuated pro-inflammatory cytokines expression at 7 days following SCI.

The qRT-PCR assay of IL-1β and iNOS mRNA expression at 7 days following injury, and respective statistical analysis. N = 5 rats per group. Values represent the mean ± SD, **p* < 0.05, ***p* < 0.01, ****p* < 0.001 according to ANOVA.

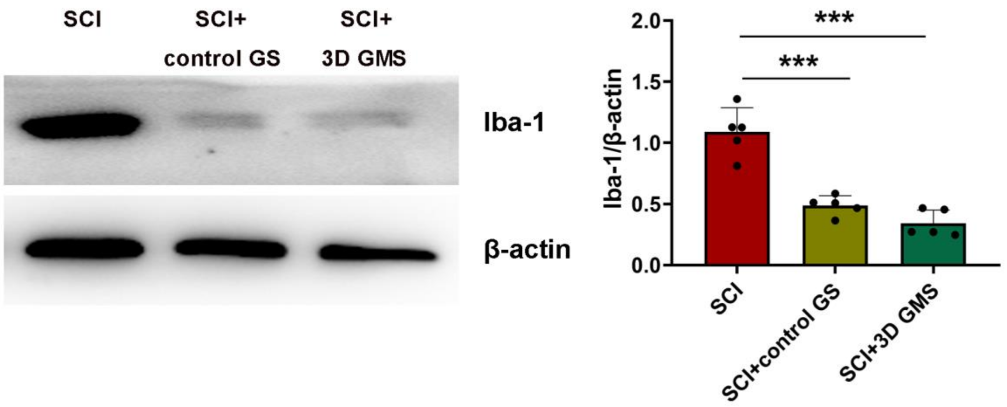

Figure S11. Western blot assay of Iba-1 protein expression.

Western blot assay of Iba-1 protein expression in the three groups, and respective statistical analysis. N = 5 rats per group. Values represent the mean ± SD, ****p* < 0.001 according to ANOVA.

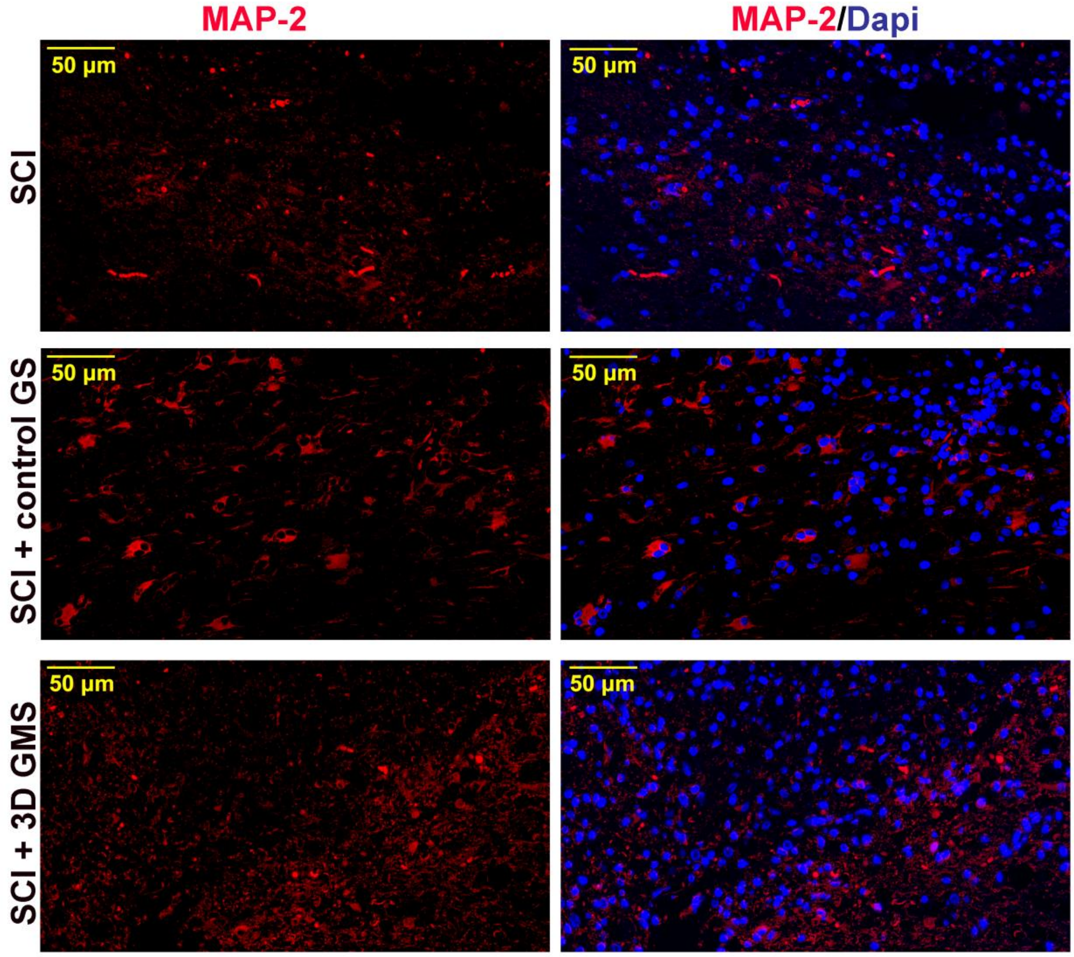

Figure S12. 3D GM scaffolds boost regeneration of nerve.

Representative images of MAP-2 (red) immunohistochemical staining at 28 days after injury in spinal cord lesion areas. All cell nuclei were counterstained with Dapi (blue). Scale bar=50 μm.

Supplementary Video

Video S1. Movement of rat's right hind limb in SCI group.

Video S2. Movement of rat's right hind limb in SCI+control GS group.

Video S3. Movement of rat's right hind limb in SCI+3D GMS group.

Supplementary Table

Table S1. Reagent in this study

Company	Description	Catalog number
CWBIO (Haimen, Jiangsu, China)	BCA protein assay kit	CW0014S
Zhongshan Golden Bridge Biotechnology (Beijing, China)	anti-β-actin	TA-09
Zhongshan Golden Bridge Biotechnology (Beijing, China)	Peroxidase-conjugated goat anti-mouse IgG	ZB-2305
Zhongshan Golden Bridge Biotechnology (Beijing, China)	Peroxidase-conjugated goat anti-rabbit IgG	ZB-2301
Abcam (Cambridge, MA, USA)	Rabbit Polyclonal anti-MAP-2 antibody	Ab32454
Cell Signaling Technology, Inc. (Boston, MA, USA)	Rabbit Monoclonal anti-AKT antibody	9272S
Cell Signaling Technology, Inc. (Boston, MA, USA)	Rabbit Monoclonal anti-p-AKT antibody	9271S
Cell Signaling Technology, Inc. (Boston, MA, USA)	Rabbit Monoclonal anti-ERK antibody	9102S
Cell Signaling Technology, Inc. (Boston, MA, USA)	Rabbit Monoclonal anti-p-ERK antibody	9101S

Cell Signaling Technology, Inc. (Boston, MA, USA)	Rabbit Monoclonal anti-NF antibody	2837S
Abcam (Cambridge, MA, USA)	Rabbit Polyclonal anti-Iba-1 antibody	Ab178847
Proteintech Group (Rosemont, IL, USA)	Mouse Monoclonal anti-GFAP antibody	60190-1-Ig
Proteintech Group (Rosemont, IL, USA)	Rabbit Polyclonal anti-CD44 antibody	15675-1-AP
Millipore Corporation (Billerica, MA, USA)	Enhanced chemiluminescence and PVDF membranes	No.IPVH00010
Roche Diagnostics Gmbh (Indianapolis, IN, USA)	PhosSTOP phosphatase inhibitor	P1082
Beyotime Institute of Biotechnology (Jiangsu, China)	RIPA	P0013B
Beyotime Institute of Biotechnology (Jiangsu, China)	PMSF	ST506-2
Beyotime Institute of Biotechnology (Jiangsu, China)	5× loading buffer	P0015L
CWBIO (Haimen, Jiangsu, China)	TRIzon reagent	01761/20114-1

Millipore Corporation (Billerica, MA, USA)	Immobilon Western Chemiluminescent HRP Substrate	WBKLS0100
Jackson ImmunoResearch (West Grove, PA, USA)	Alexa Fluor®488-conjugated AffiniPure Goat Anti-mouse IgG (H+L)	115-545-003
Jackson ImmunoResearch (West Grove, PA, USA)	Alexa Fluor®594-conjugated AffiniPure Goat Anti-Rabbit IgG (H+L)	115-585-003
Servicebio (Wuhan, China)	FITC Tunel Cell Apoptosis Detection Kit	G1501-50T
Servicebio (Wuhan, China)	Tris Buffered Saline (TBS) powder	G0001
Servicebio (Wuhan, China)	Phosphate Buffered Saline (PBS) powder	G0002
Solarbio (Beijing, China)	PAGE Pre-solution	A1010
TOYOBO (Shanghai, China)	ReverTra Ace Qpcr RT Kit	FSQ-101

Table S2. Swelling characteristics of 3D gelatin microsphere scaffolds

**Time (Hour)**	**Volume change from 36 mm^3**	**Volume change from 18 mm^3**	**Volume change from 8 mm^3**

0	36.62	18.31	7.67
0.5	81.36	54.11	23.37
1	120.03	64.45	30.06
2	151.72	80.70	30.00
4	154.42	100.68	38.68
8	144.21	88.07	37.89
12	144.21	89.87	35.83

Table S3. Degradation characteristics of 3D gelatin microsphere scaffolds

Time (Days)	Diameter 1 (mm)	Diameter 2 (mm)	Height (mm)	Weight (g)	Volume (mm^3)	Degradation Curve (%)
1	10.23	10.01	3.35	0.2308	269.474	100.00
2	9.77	9.45	2.9	0.2179	210.337	78.05
3	9.37	9.17	2.81	0.2109	189.647	70.38

4	8.75	8.59	2.56	0.1907	151.142	56.09
6	8.56	8.07	2.38	0.1766	129.234	47.96
8	8.44	7.94	2.31	0.1687	121.691	45.16
10	8.24	7.35	2.14	0.1183	102.121	37.90
12	7.67	6.47	1.47	0.0517	57.7122	21.42
14	6.88	5.9	0.58	0.0301	18.601	6.90
17	0	0	0	0	0	0

Degradation curve (%) = Volum (mm^3) / Swelling equilibrium volume × 100 %

Swelling equilibrium volume = Volum (Days 1) = 269.474 mm^3
